# Reconstruction of Nuclear Ensemble Approach Electronic Spectra Using Probabilistic Machine Learning

**DOI:** 10.1021/acs.jctc.2c00004

**Published:** 2022-04-28

**Authors:** Luis Cerdán, Daniel Roca-Sanjuán

**Affiliations:** Institut de Ciència Molecular, Universitat de València, València 46071, Spain

## Abstract

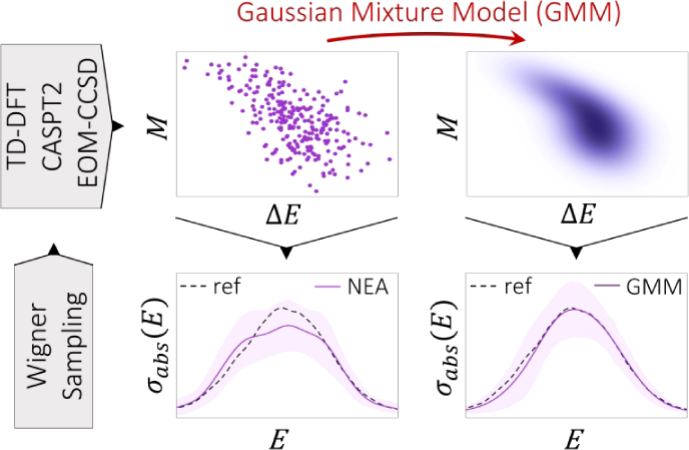

The theoretical prediction of molecular electronic spectra by means of quantum mechanical (QM) computations is fundamental to gain a deep insight into many photophysical and photochemical processes. A computational strategy that is attracting significant attention is the so-called Nuclear Ensemble Approach (NEA), that relies on generating a representative ensemble of nuclear geometries around the equilibrium structure and computing the vertical excitation energies (Δ*E*) and oscillator strengths (*f*) and *phenomenologically broadening* each transition with a line-shaped function with empirical full-width δ. Frequently, the choice of δ is carried out by visually finding the trade-off between artificial vibronic features (small δ) and over-smoothing of electronic signatures (large δ). Nevertheless, this approach is not satisfactory, as it relies on a subjective perception and may lead to spectral inaccuracies overall when the number of sampled configurations is limited due to an excessive computational burden (high-level QM methods, complex systems, solvent effects, etc.). In this work, we have developed and tested a new approach to reconstruct NEA spectra, dubbed GMM-NEA, based on the use of Gaussian Mixture Models (GMMs), a probabilistic machine learning algorithm, that circumvents the phenomenological broadening assumption and, in turn, the use of δ altogether. We show that GMM-NEA systematically outperforms other data-driven models to automatically select δ overall for small datasets. In addition, we report the use of an algorithm to detect anomalous QM computations (outliers) that can affect the overall shape and uncertainty of the NEA spectra. Finally, we apply GMM-NEA to predict the photolysis rate for HgBrOOH, a compound involved in Earth’s atmospheric chemistry.

## Introduction

The accurate and reliable prediction of absorption and emission spectra of molecular compounds by means of quantum mechanical (QM) computations is fundamental for the understanding and discovery of many photophysical and photochemical processes in which an experimental determination becomes unfeasible and/or cannot provide insights into the underlying physics.^1−9^ The simulation of spectral shapes from first principles taking into account all relevant broadening mechanisms is an extremely challenging task, both from theoretical and computational points of view, as it entails the simulation of excited-state quantum molecular dynamics and subsequent calculation of the auto-correlation function between the ground-state wave function and the time-dependent excited-state one.^10−12^ A more affordable (time-independent) strategy is the so-called Nuclear Ensemble Approach (NEA).^13,14^ This method relies on generating a representative ensemble of nuclear geometries around the equilibrium structure and computing (to the desired QM accuracy) their vertical excitation energies (Δ*E*) and oscillator strengths (*f*) for all pertinent states. Each of these transitions is *phenomenologically broadened* by assigning a Gaussian or Lorentzian line shape centered at Δ*E*, with an empirical full-width δ and with an area proportional to the corresponding *f*. The average of these multiple Gaussians builds up the electronic spectrum (see details below). In this sense, the larger the number of geometries is, the more accurate the spectrum *reconstruction* becomes. This method has attracted significant attention in the last decade, as it allows to predict reliable electronic absorption and emission spectra without a prohibitive computational burden.^15−29^

Unfortunately, the total number of sampled geometries onto which to perform QM computations may be limited to a few hundred, in the best cases, in situations requiring an expensive computational power, for example, when resorting to high-level QM methods (EOM-CCSD, CASSCF/CASPT2, etc.) and/or treating with more complex systems (large number of excited electronic states, spin–orbit coupling, large molecules, solvent effects, etc.). This limitation in the amount of data may lead to inaccuracies in the reconstructed spectra if the line-width δ for each of the Gaussians is not chosen properly. In this sense, it should be chosen so that a trade-off between artificial vibronic features (small δ) and over-smoothing of electronic signatures (large δ) is attained. Frequently, the choice of empirical line-width δ is carried out by trial and error through the visual inspection of the reconstructed spectra and finding the compromise between under- and over-smoothing. Nevertheless, this approach is not satisfactory, as it relies on a subjective perception. Accordingly, there is an effervescent interest in finding objective criteria to adequately reconstruct the electronic NEA spectra for small datasets.^23,27,28,30,31^ In this sense, two different schools of thought, both based on data-driven methods, can be currently found: either training a supervised machine learning (ML) algorithm with the available geometries to later predict Δ*E* and *f* for a large amount of sampled geometries so that the choice of δ is not so critical as long as it is sensibly chosen^30,31^ or resorting to unsupervised ML models to extremely fine tune δ.^23,27,28^

The last years have witnessed a surge in the application of ML and deep learning (DL) techniques to solve problems in excited-state chemistry with high success.^32,33^ For the prediction of electronic spectra, in particular, an ML or DL algorithm is trained to act as a surrogate for the function mapping the molecular structure space to the Δ*E* and/or *f* spaces. In other words, the ML/DL models are used as non-linear regression functions relating a molecular input, **X**, to a quantum chemical output, *Y*. In such a way, when the model is presented with a new geometry, it can predict the values for Δ*E* and/or *f* without resorting to expensive QM computations. Neural networks (NNs) such as SchNet^34−36^ have shown great potential in predicting absorption spectra, even enabling transferability in the chemical space (training the NN with a set of molecules, predicting the properties for a different set).^37^ A notorious drawback of NNs in general, and SchNet in particular, is that they usually require thousands of training instances (e.g., sampled geometries for the NEA spectra),^37−39^ precluding its use for small datasets. In these cases, ML kernel-based methods have been proposed as suitable alternatives to NNs.^32,33^ In this family of algorithms, the molecular input features (**X**) are mapped, by means of a non-linear function (kernel), into a higher-dimensional space where the transformed features are linearly related to the quantum chemical output *Y* (Δ*E* and/or *f* for NEA spectra). Among them, the KREG model has been successfully used to reconstruct NEA spectra when the number of quantum chemical computations is limited.^30,31^ This ML model relies on Kernel Ridge Regression with a Gaussian kernel function and ridge regularization and uses the normalized inverted internuclear distances as molecular features/descriptors (**X**).^40^ A few hundreds of training instances (Wigner sampled geometries) suffice to train the KREG model, enabling the prediction of Δ*E* and *f* for thousands of unseen geometries without additional computational burden, thus affording satisfactory NEA spectral reconstructions.^30,31^

A different paradigm in ML is the so-called unsupervised learning, where the algorithm is not a non-linear regression function relating **X** to *Y* but a model that looks for data structures *hidden* within a dataset (**X** or *Y*). As with supervised ML, unsupervised ML has been already applied to the assessment of excited-state chemistry problems.^32,33^ The approaches which reconstruct the electronic NEA spectra for small datasets extremely fine tuning the bandwidths δ are based on this paradigm.^23,27,28^ Focusing exclusively on the available computed Δ*E* and *f*, these studies infer the optimal δ for each transition applying conventional techniques on Kernel Density Estimation (KDE), a nonparametric model to estimate the probability density function (PDF) underlying a random variable.^41^ In this case, both the sample size *n* (number of geometries) and the distribution of the pairs {Δ*E*_*i*_,*f*_*i*_}_*i*=1,...,*n*_ determine the optimal δ. One of the advantages of this approach with respect to the KREG model or NNs is that first, it renders a different optimal δ for each transition and, second, that it performs well even for datasets with less than a hundred of geometries. In fact, it has been recently shown that the optimal choice of the nuclear ensemble geometries used for the quantum chemistry calculations enables the reliable reconstruction of NEA spectra with just a few tens of geometries.^23^

Although both approaches to improve the reconstruction of NEA spectra lead to broadly satisfactory results, all the models reported to date still rely on the use of the phenomenological broadening for each of the transitions underpinning the NEA approach. To eliminate this dependency and the selection of a bandwidth altogether, we report in this article a new approach based on the use of Gaussian Mixture Models (GMMs), an unsupervised ML algorithm commonly used for clustering, classification, and density estimation tasks.^42,43^ We compare the performance of this model with that of the automatic δ selection models based on KDE and the regression-based KREG model. With this aim, we introduce a new metric to make spectral reconstruction comparisons and propose its use as a stopping criterion in an *active learning* strategy. In addition, we report, for the first time, the use of an algorithm to detect anomalous QM computations (outliers) that can affect the overall shape and uncertainty of the NEA spectra. Finally, we apply the new model to the prediction of the photolysis rate for a compound of interest in atmospheric chemistry.

## Methodology

### NEA Spectra, Discrete Version

The theoretical framework for the generation of absorption spectra is based on a semiclassical description of the light/matter interaction, where the electromagnetic fields are treated as classical quantities, obeying Maxwell’s equations, whereas the matter is described by means of QM averages.^44^ Within time-dependent perturbation theory, under the electric dipole and Born–Oppenheimer approximations and the application of a Monte Carlo (MC) nuclear ensemble sampling, the absorption cross section for a single transition (σ_abs,*n*_(*E*)) as a function of photon energy *E* is given by^14^
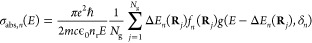
1where *e* and *m* are the charge and mass of the electron, respectively, *c* is the speed of light in vacuum, ℏ is the reduced Planck constant, ϵ_0_ is the vacuum permittivity, *n*_r_ is the refractive index at the spectral region of the transitions (no optical dispersion assumption), and *N*_g_ is the number of sampled geometries. For each sampled geometry with nuclear coordinates **R**_*j*_, *f*_*n*_ and Δ*E*_*n*_ are, respectively, the oscillator strength and the vertical energy of the transition from the ground state to the *n*-th excited state. The transition line-shape *g*(*E* – Δ*E*_*n*_(**R**_*j*_), δ_*n*_) is given by using a normalized Gaussian
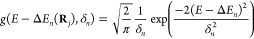
2where δ_*n*_ is the full-width and is usually determined phenomenologically. The full NEA spectrum cross-section (σ_abs_(*E*)) is constructed through the incoherent contribution (sum) of all possible excited states *N*_s_ as
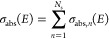
3

The statistical error (confidence intervals, CIs) associated to the MC sampling can be inferred either using directly the standard error (assuming asymptotic normality)^14,25^ or using a re-sampling technique such as bootstrap.^26^ As normality is not granted either on Δ*E* or on *f* (see Figure S1), it is statistically more robust to use a bootstrap re-sampling.^45^ In this procedure, a large number *B* of new samples (bootstrap replicas) are generated by randomly sampling with replacement *N*_g_ pairs {Δ*E*_*n*_, *f*_*n*_} from the *N*_g_ available ones. For each bootstrap replica, the NEA spectrum for each state is computed using eq 1. Accordingly, for each energy/wavelength, there will be a distribution of cross sections (σ̂_n_^*^(*E*)). Assuming a *percentile bootstrap*, the lower (*l*) and upper (*u*) CIs are obtained as
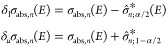
4where σ̂_α/2_^*^ and σ̂_1−α/2_^*^ are the quantiles α/2 and 1 – α/2, respectively, with α the confidence level of the distributions σ̂*. In this article, we have selected a 95% CI (α = 0.05), and thus, the lower and upper CIs are given by the quantiles 2.5 and 97.5%, respectively. Finally, the lower and upper CIs for the full NEA spectrum are given by
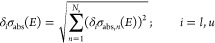
5

### Automatic Selection of Empirical Broadening (auto-δ)

Let  be a set of *n*-independent and identically distributed (iid) events of a *p*-dimensional random variable **X** = (*X*_1_, ..., *X*_*p*_) drawn from an unknown, unobservable joint PDF *f*_**X**_(**x**). Finding an estimate *f̂*_**X**_(**x**) from sample  is of paramount importance in statistics and probability theory.^46^

KDE is a nonparametric model to estimate *f*_**X**_(**x**) that makes almost no assumptions about the underlying distribution. In KDE, each observation in the sample contributes locally to the PDF through a smooth symmetric function (Kernel). Restricting ourselves to the univariate case (*p* = 1), the KDE estimator is given by^46,47^
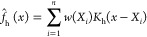
6where *w*(·) is a weight function, and *K*(·) is a kernel function, the characteristic bandwidth *h* of which controls the estimate smoothness. If all observations have the same weight, then *w*(*X*_*i*_) = 1/*n*, and one recovers the standard KDE.^46^ A common choice for the kernel function is the normalized Gaussian
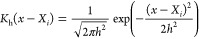
7

The bandwidth *h* should be chosen so that a trade-off between noise (small *h*) and over-smoothing (large *h*) is attained. A rule of thumb to choose the optimal bandwidth is given by^47^
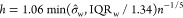
8where σ̂_w_ and IQR_w_ are the weighted versions of the sample standard deviation and sample interquartile range, respectively. This bandwidth minimizes the mean integrated squared error (MISE) between the real underlying PDF *f*_*X*_(*x*) and the KDE *f̂*_h_(*x*) when *X* is close to normally distributed.^41^

Now, let us connect KDE with the NEA spectra. Notice that eqs 1 and 3 can be recast as
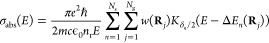
9where *w*(**R**_*j*_) = Δ*E*_*n*_(**R**_*j*_)*f*_*n*_(**R**_*j*_)/*N*_g_. The summation over geometries *j* is exactly eq 6, meaning that the problem of NEA electronic spectral reconstruction is formally analogous to KDE. Accordingly, the empirical bandwidth that optimizes the shape of each band in the electronic spectrum is δ_*n*,opt_ = 2*h*, with *h* given by eq 8. In this sense, for each transition *n*, one has to compute the weights *w*(**R**_*j*_) for the KDE and, with them, the weighted standard deviation σ̂_w_ and weighted IQR_w_ of the Δ*E*_*n*_. Thus, we have found a straightforward method that allows determining in a band-wise fashion the best empirical bandwidths using a data-driven strategy. We will refer to this method as auto-δ.

Incidentally, Sršeň et al.^23,27^ reported a slightly different version of this method based on original Silverman’s rule of thumb,^41^ where eq 8 only considers the weighted standard deviation σ̂_w_ and assumes an effective sample size *n*_eff_. This method implies a normal distribution for the data (Δ*E*), an assumption that cannot be always guaranteed. The incorporation of the IQR into eq 8 allows for gentle deviations from normality, hence being a more robust estimator. Nevertheless, both methods will provide analogous results. Furthermore, Fehér et al.^28^ have just reported a similar, but slightly more sophisticated, method to find the optimal bandwidths through an optimization problem. These authors make as well the connection between eq 1 and KDE and find the bandwidth minimizing simultaneously the MISE between the originally computed *f* values and those “predicted” by the kernel function and the leave-one-out cross-validation error. In contrast, the bandwidth *h* (or δ_*n*_) given by eq 8 has been shown to minimize the MISE between the real underlying PDF *f*_*X*_(*x*) and the KDE *f̂*_h_(*x*) when *X* is close to normally distributed,^41^ as it is the case with Δ*E* (Figure S1). Thus, the three methods will provide similar results.

### Complete Elimination of Empirical Broadening (GMM-NEA)

Even when we have managed to establish a methodology to avoid the manual selection of δ, the fact that it is an artificial or phenomenological broadening still remains. To eliminate this artifact, we report a new approach based on the use of GMMs.^42,43,46,48,49^ From a conceptual point of view, GMMs are probabilistic models that assume that all the data points in a dataset are generated from a finite mixture of normal distributions with unknown parameters. In the context of clustering, a common unsupervised ML task to find groups or clusters of points sharing common characteristics (*e.g.*, customers, patients, genes, voices, images, etc.), each component of the GMM would represent a cluster. Furthermore, once the cluster structure is found, GMMs can serve as classifiers to assign new observations to its corresponding cluster. However, what is more important in the context of this work is that GMMs are very powerful density estimators, this is, they are algorithms that allow inferring the continuous probability distribution underlying a discrete distribution of points.^42,43^

From a mathematical perspective, GMMs rely on the fact that any multivariate PDF supported in the real plane can be decomposed into a finite sum (mixture) of normal distributions.^42,43^ Let  be a set of *n* iid events of a bidimensional random variable **X** = (*X*_1_, *X*_2_) drawn from an unknown joint PDF *f*_**X**_(**x**). Then, the joint PDF can be modeled as
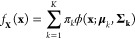
10where each bivariate Gaussian PDF ϕ(**x**;**μ**_*k*_,**Σ**_**k**_) has its own vector of means **μ**_*k*_ = (μ_*k*,1_, μ_*k*,2_) and the covariance matrix **Σ**_*k*_ = (σ_*k*,1_^2^,ρ_*k*_σ_*k*,1_σ_*k*,2_;ρ_*k*_σ_*k*,1_σ_*k*,2_,σ_*k*,2_^2^), where σ_*k*,1_^2^ and σ_*k*,2_^2^ are the variances of the mixture covariates, and ρ_*k*_ is the correlation coefficient. The parameters π_*k*_ are the mixing coefficients, weights, or priors for each component of the mixture and must fulfill the conditions 0 ≤ π_*k*_ ≤ 1 and ∑π_*k*_ = 1.

**Figure 1:**
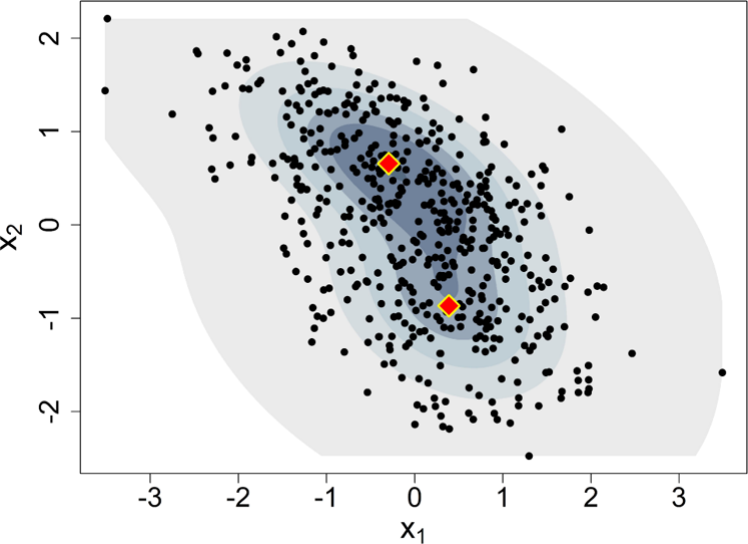
Sample of 500 observations (points) drawn from an unknown distribution and the estimated joint PDF (shaded contours) assuming *K* = 2 components for the GMM model. The diamonds mark the location of the mixture means.

The mixture parameters **Ψ** = {π_1_, ..., π_*k*–1_, **μ**_1_, ..., **μ**_*k*_, **Σ**_1_, ..., **Σ**_*k*_} must be chosen so that they maximize the log likelihood of set  having been drawn from mixture eq 10. A powerful iterative method for estimating the mixture parameters locally maximizing the likelihood is the *Expectation-Maximization* algorithm or *EM* algorithm.^46^ First, some initial values for the means, covariances, and mixing coefficients are chosen. In the *expectation* step, or E step, the current parameter estimates are used to evaluate the posterior probabilities, or responsibilities, of a given observation to belong to a given mixture component. In the *maximization* step, or M step, these responsibilities are used as *weights* to update the means, covariances, and mixing coefficients. Finally, the log likelihood is computed for these new estimates. These steps are repeated until either the parameters or the log likelihood has converged. The interested reader can find the expressions to compute the likelihoods, responsibilities, and updated parameters elsewhere.^46^Figure 1 shows an example of a sample of a bidimensional random variable drawn from an unknown distribution and the joint PDF of the underlying distribution estimated using GMMs.

A key aspect of mixture models, in general, and GMMs, in particular, is *model selection* or how many components *K* to include in the mixture and which constraints  to apply to the covariance matrices (spherical, diagonal, or ellipsoidal). The most common model selection procedure in the context of GMMs consists of maximizing the Bayesian Information Criterion (BIC), which is given by
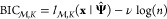
11where  is the log likelihood of model  with estimated parameters **Ψ̂**, *n* is the sample size, and ν is the number of estimated parameters. Thus, the pair  maximizing  is selected. The BIC, like other model selection criteria, looks for a compromise between precision (small log likelihood) and model complexity/simplicity (small number of parameters). The term ν  log(*n*) in eq 11 acts as a regularization term that penalizes models which are too complex and thus avoids overfitting. This means that even when a more complex GMM could be needed to exactly model the distribution, the BIC could suggest a simpler GMM. In the NEA context, the spectra generated with GMMs (vide infra) could be slightly smoother than the real ones.

Now, let us connect GMMs with the NEA spectra. For reasons that will transpire later on, eq 1 is recast as
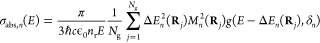
12where *f* has been expressed as transition dipole moments using the relation *M*^2^ = 3ℏ^2^*e*^2^*f*/2*m*Δ*E*. Notice that the summation is nothing but the discrete mean or expected value  of the function ς(Δ*E*_*n*_,*M*_*n*_) = Δ*E*_*n*_^2^*M*_*n*_^2^*g*(*E* – Δ*E*_*n*_,δ_*n*_). The way in which NEA is constructed, each pair {Δ*E*_*n*_(**R**_*j*_), *M*_*n*_(**R**_*j*_)} is equiprobable, thus the factor 1/*N*_g_ in front of the summation.^14^ Nevertheless, based on physical grounds, Δ*E*_*n*_ and *M*_*n*_ are continuous random variables, and not all pairs {Δ*E*_*n*_, *M*_*n*_} are equally probable. For a continuous random variable **X**, the expected value of a function *g*(**X**) is given by the Lebesgue integral , where *f*_**X**_(**x**) is the joint PDF of **X**. Applying this same principle, discrete eq 12 can be turned into a continuous version given by
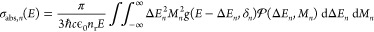
13where  is the probability of finding the pair {Δ*E*_*n*_, *M*_*n*_}. In other words, the unknown joint PDF *f*_**X**_(**x**) with **X** = (Δ*E*_*n*_, *M*_*n*_). However, we have just seen that an unknown PDF can be estimated using GMMs, and then, we can make the substitution (cf. eq 10)
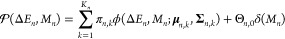
14where **μ**_*n*,*k*_ = (μ_1,*n*,*k*_, μ_2,*n*,*k*_) and **Σ**_*n*,*k*_ = (σ_1,*n*,*k*_^2^,ρ_*n*,*k*_σ_1,*n*,*k*_σ_2,*n*,*k*_;ρ_*n*,*k*_σ_1,*n*,*k*_σ_2,*n*,*k*_,σ_2,*n*,*k*_^2^). The subindices 1 and 2 make reference to the corresponding variable Δ*E*_*n*_ and *M*_*n*_, respectively. Notice that an additional term has been included to take into account that for some transitions and geometries, *M*_*n*_ = 0 (forbidden transition). Thus, Θ_*n*,0_ is the probability of *M*_*n*_ being exactly 0, and δ(*M*_*n*_) is the Dirac delta distribution. It is important to stress that the addition of this term implies that ∑_*k*_π_*n*,*k*_ ≠ 1, but ∑_*k*_π_*n*,*k*_ = 1 – Θ_*n*,0_. The estimate Θ̂_*n*,0_ is obtained by simply computing the proportion of sampled geometries with *M*_*n*_ = 0. Now, the substitution of eq 14 into eq 13 yields
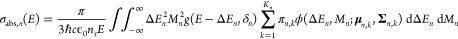
15

Notice that upon applying the above-mentioned transformation, the explicit dependency on the molecular geometry **R**_*j*_ vanishes, and it is not required anymore, as it is implicitly contained within  or its GMM model.

In any case, the dependency on the empirical linewidth δ_*n*_ still must be removed. To do so, one must resort to the nice properties of the Gaussian function. In the limit where δ_*n*_ → 0, one has
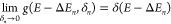
16where δ(*E* – Δ*E*_*n*_) is the Dirac delta function centered at Δ*E*_*n*_ = *E*. Thus, taking the limit of eq 15 when δ_*n*_ → 0, using relation eq 16, and applying the Dirac delta function property ∫_–∞_^∞^*f*(*x*)δ(*x* – *a*) d*x* = *f*(*a*) yield the simplified expression
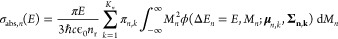
17

Remarkably, this expression does not depend anymore on the empirical linewidth δ_*n*_. This equation can be simplified further by noting that a joint PDF evaluated at a given value of one of the covariates can be factorized as *f*_**X**_(*x*_1_ = *x*, *x*_2_) = *f*_*X*_1__(*x*)*f*_*X*_2_|*X*_1_=*x*_(*x*_2_), where the first and second terms on the right-hand side are the marginal and conditional PDFs, respectively.^50^ For the case of normal distributions, both PDFs follow Gaussian functions, and one finds the relation
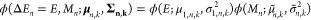
18where μ̃_*n*,*k*_ and σ̃_*n*,*k*_^2^ are given by^50^
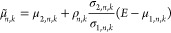
19
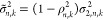
20

Plugging in eqs 18–20 into eq 17 leads to
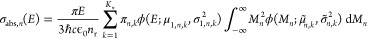
21

The integral in eq 21 is the second-order moment or expectation value of *M*_*n*_^2^ under the Gaussian distribution ϕ(*M*_*n*_;μ̃_*n*,*k*_,σ̃_*n*,*k*_^2^), which is exactly solvable and equals μ̃_*n*,*k*_^2^ + σ̃_*n*,*k*_^2^.^51^ Accordingly, eq 21 is simplified to
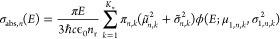
22where μ̃_*n*,*k*_ and σ̃_*n*,*k*_^2^ are given by eqs 19 and 20, respectively.

The full NEA spectrum (σ_abs_(*E*)) is constructed again as the incoherent sum of all possible transitions (eq 3). With the foregoing procedure, we have obtained a continuous version of the NEA spectra which does not depend anymore on empirical bandwidths. In this sense, eq 22 constitutes the main result of this article. This method, based on the use of unsupervised ML, will be referred to in subsequent sections as GMM-NEA.

From a practical point of view, to refine the NEA spectra using GMM-NEA, one should proceed, for each transition independently, as follows: Estimate the proportion Θ̂_*n*,0_ of sampled geometries with *M*_*n*_ = 0 and remove them from the dataset. Using the remaining geometries (pairs {Δ*E*_*n*_, *M*_*n*_}), carry out a model selection to find GMM constraints  and number of mixtures *K*_*n*_ that maximizes the BIC (eq 11). Retrieve the estimated means (μ_*n*,*k*,1_, μ_*n*,*k*,2_), variances (σ_*n*,*k*,1_^2^, σ_*n*,*k*,2_^2^), correlation coefficients (ρ_*n*,*k*_), and weights (π_*n*,*k*_) associated to each of the mixtures of the optimized GMM model. Multiply the estimated weights by (1 – Θ̂_*n*,0_) so that we guarantee that ∑_*k*_π_*n*,*k*_ = 1 – Θ̂_*n*,0_. Finally, substitute the estimated parameters (means, variances, correlations, and rescaled weights) into eqs 19 and 20 and reconstruct the electronic spectrum using eq 22.

The statistical error (CIs) associated with the GMM-NEA spectra must be inferred using bootstrap. In this case, for each bootstrap replica and transition, a GMM model with the number of mixtures *K*_*n*_ and constraints  maximizing the BIC in the original dataset (zeros removed) is fitted. The resulting GMM parameters are used to reconstruct each bootstrap replica transition spectrum using eq 22. Finally, the lower and upper CI for each transition and the full NEA spectrum is computed with eqs 4 and 5, respectively. Again, we have selected a 95% CI (α = 0.05) and *B* = 999 bootstrap replicas.

### Outlier Detection

The presence of extreme or anomalous events that significantly differ from the main bulk of the data may distort any statistical procedure applied upon a multivariate dataset. Thus, the detection of this so-called outliers, which may or may not be real anomalous events, is of paramount importance for ML algorithms in general and GMMs in particular.^52^ Among the many algorithms for outlier/anomaly detection, we will use the false discovery rate (FDR) method in combination with the squared Mahalanobis distance *D*_M_^2^.^53^ This method has been chosen because it is very common, it has been covered by extensive literature,^53−56^ it is easy to interpret, and it allows one to have relative control on the percentage of false positives.

The Mahalanobis distance measures the distance of any given observation **x** = (*x*_1_, *x*_2_, ..., *x*_*p*_) in a *p*-dimensional space to a given distribution of *n* samples as
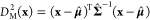
23where **μ̂** = (μ̂_1_, μ̂_2_, ..., μ̂_*p*_) is the sample mean vector, and **Σ̂** is the sample covariance matrix. Accordingly, one can easily see that the possible outliers of the distribution will display large Mahalanobis distances. It remains to be seen how large is *large*. For a multivariate normally distributed random variable **X**, it can be shown that *D*_M_^2^(**x**) ∼ χ_*p*_^2^, that is, it follows a chi-squared distribution with *p* degrees of freedom. Thus, the possible outliers can be identified, with an FDR below *q* ∈ (0, 1], as follows: Compute the distances *D*_M_^2^(**x**) for all observations in the sample. Compute their corresponding *p*-values *p*_1_, ..., *p*_*n*_ assuming that they follow a χ_*p*_^2^ distribution. Label as outliers those observations with *p*-values below ρ_*i*_*q*/*n*, where ρ_*i*_ is the rank of the *i*-th *p*-value. It can be proved that this procedure guarantees that the proportion of false outlier detections is below *q*.^54^

There are some caveats to this method. The condition *D*_M_^2^(**x**) ∼ χ_*p*_^2^ is exactly valid only for normally distributed random variables, but it has been shown that it can be used for non-normally distributed variables as it is the case with Δ*E*_*n*_ and dipole moments *M*_*n*_ (Figure S1). In addition, the presence of outliers may influence the estimates for the mean vector **μ̂** and covariance matrix **Σ̂**, thus modifying the distribution of *D*_M_^2^(**x**) and, in turn, the detection of outliers itself. For this reason, it is fundamental to use robust estimates for the location and scatter of data immune to the presence of outliers. Among the many robust estimates found in the literature,^53,56^ we have used as the robust location estimate **μ̂**_R_ the median instead of the mean and as the robust scatter estimate the covariance matrix computed using the robust estimate **μ̂**_R_, that is, **Σ̂**_R_ = 1/(*n* – 1)**M**_**X**_**M**_**X**_^T^, where **M**_**X**_ is the matrix of observations centered at the median. Finally, to guarantee a conservative selection of outliers, we set *q* = 0.001, that is, we forced less than 0.1% false outlier detections.

### Relative Integral Change

A conundrum posed by the generation of NEA electronic spectra refined by any of the above-described methods is to assess the goodness of the reconstruction and to decide how many geometries to compute and use in the reconstruction to find a compromise between accuracy and computational burden. This can be done by visual inspection of the generated spectra (subjective way) or by using quantitative metrics (objective way). Xue et al. introduced the relative integral change (RIC),^30^ which measures the relative difference between the reconstructed spectrum σ_R_(*E*) and the expected/target spectrum σ_T_(*E*) as
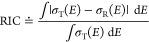
24

In this sense, if the reconstruction is perfect, then RIC = 0. Although this metric has proven useful, its results may be misleading, as it “over-rewards” a good reconstruction of the strongest bands, while neglecting the reconstruction of the weakest bands. Accordingly, in this work, we will use a band-wise RIC, or bRIC, computed as
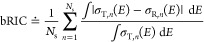
25where σ_R,*n*_(*E*) and σ_T,*n*_(*E*) are, respectively, the reconstructed and target electronic spectra for band *n*. In this way, all bands contribute equally irrespective of their strength.

The metrics RIC or bRIC as defined above are useful if there is a target spectrum, but that is not the situation in real scenarios. In these cases, it is better to resort to an “active learning” strategy, where an extra set of samples (*batch*) must be computed if a certain criterion is not met. In this work, we propose as criterion a sequential version of bRIC, defined as
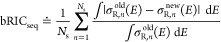
26where σ_R,*n*_^old^(*E*) and σ_R,*n*_^new^(*E*) are, respectively, the reconstructed electronic spectra for band *n* computed without and with the extra batch of data. Thus, bRIC_seq_ would tend to 0 as the number of added batches is increased. In this sense, the “active learning” should be stopped when this metric falls below a given threshold value. Be aware that a change in batch size should be accompanied by a change in this threshold.

### Datasets and Computational Details

The main body of the workload in this publication will be carried out using freely available data on Δ*E* and *f* computed for benzene,^30^ an acridine derivative (Comp2),^57^ and an acridophosphine derivative (Comp3)^57^ using TD-DFT as the QM framework. The interested reader is referred to the original publications for the computational details. For benzene, there are pairs {Δ*E*, *f*} for 10 different transitions and 50,000 geometries, whereas for Comp2 and Comp3, there are data for 30 transitions and 2000 geometries.

In addition, values for Δ*E* and *f* for the uracil nucleobase OH radical (U6OH radical)^29^ and the HgBrOOH atmospheric compound,^58^ both previously reported by our group, have been used to test the methodologies developed in the current work. For the U6OH radical, there are pairs {Δ*E*, *f*} for 9 different transitions and 100 geometries, whereas for HgBrOOH, there are data for 79 transitions and 200 geometries. They were obtained by using multiconfigurational quantum chemistry, in particular, the CASPT2 method. Spin-free states and spin–orbit states were used, respectively, for the U6OH radical and HgBrOOH (see the references for details).

All the methods described in this work have been implemented in R. In particular, we have used library *mclust* version 5,^59^ a very powerful and versatile package that allows modeling data with GMMs using the EM algorithm for classification, clustering, and density estimation. This package allows performing an automatic model selection (maximization of the BIC) using a pool of different covariance structures (model constraints ) and different numbers of mixture components *K*. A study on the computation cost of auto-δ and GMM-NEA can be found in the Supporting Information and Figure S2.

## Results and Discussion

In the remaining article, a comparison between auto-δ and GMM-NEA electronic spectrum reconstructions and a quantification of the differences will be presented. As a reminder, the auto-δ spectra are calculated by means of the conventional NEA expression (eq 1) but with an empirical broadening automatically determined by using a data-driven approach (eq 8). In contrast, for the GMM-NEA spectra, a GMM is fitted to the data, and the fitted parameters are used to calculate the spectra with eq 22. This section is organized as follows: Using benzene, Comp2, and Comp3, we start by presenting a visual inspection of the reconstructed spectra and its similitude to the target spectra and assessing the influence of the sample size and bias on the reconstruction accuracy (RIC and bRIC). We will unveil the effects of outliers on the U6OH radical and, finally, will compare the photolysis rates obtained for HgBrOOH using auto-δ and GMM-NEA.

### auto-δ Versus GMM-NEA: A Visual Analysis

**Figure 2:**
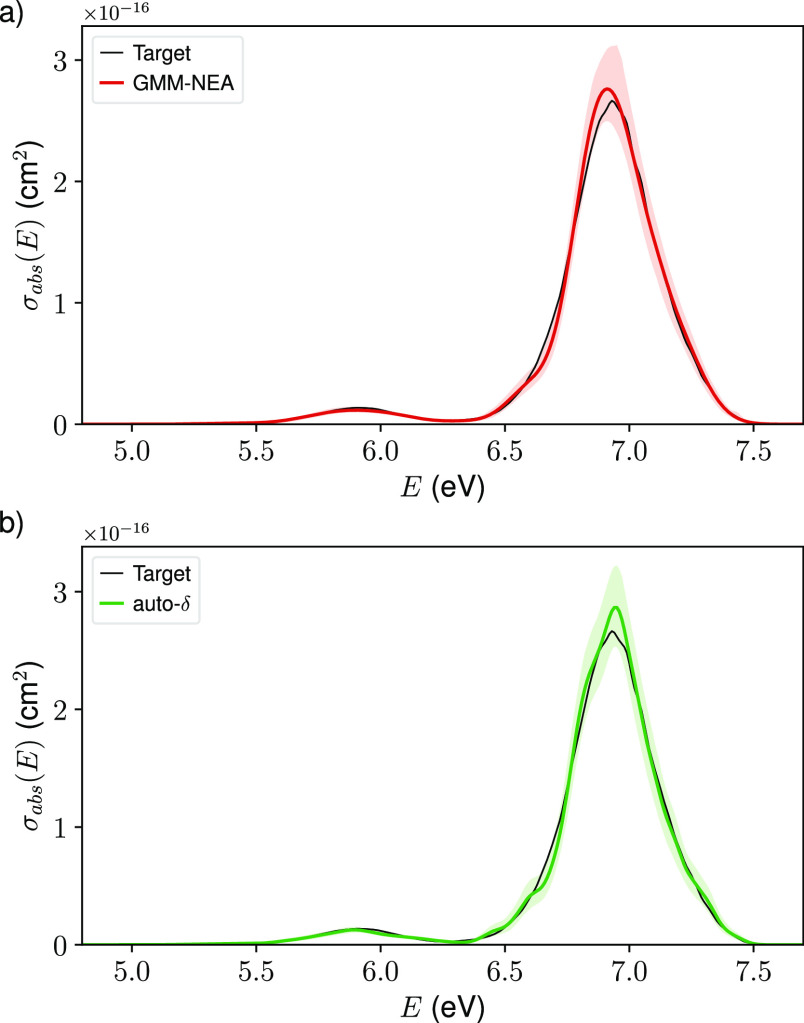
Electronic absorption cross-section spectrum of benzene reconstructed from 250 geometries using (a) GMM-NEA and (b) auto-δ. The shaded areas represent the reconstruction of 95% CIs. The target spectrum (black lines) is included for comparison purposes.

For the forthcoming analysis, the target spectra were generated using auto-δ with all available geometries (50,000 for benzene and 2000 for Comp2 and Comp3). As it is clearly seen in Figure 2, both methods provide reliable reconstructions for benzene even when *trained* with only 250 geometries, finding a good balance between the avoidance of artificial “vibronic” bands (δ too narrow) and an excessive smoothing or washing out of electronic details (δ too wide). In this sense, GMM-NEA has a larger smoothing effect than auto-δ, but the uncertainty of the reconstruction is similar. This makes sense, as GMM-NEA does not use empirical bandwidths, and the electronic details are not determined by the available pairs {Δ*E*, *f*} but by their underlying distribution. Analogous results have been obtained for Comp2 and Comp3 using again 250 geometries (Figures S3 and S4).

The model parameters use to reconstruct the electronic spectra in Figure 2 are included in Table 1 (Those for Figures S3 and S4 can be found in Table S1). As it was expected (cf. eq 8), increasing the number of geometries entails a reduction in the optimal bandwidths (δ_*n*,250_ vs δ_*n*,50000_ in Table 1). Furthermore, as it has been reported before,^27,28^ the use of auto-δ unveils that every transition requires its own bandwidth. For example, notice that when using 250 geometries (δ_*n*,250_), the optimal bandwidth for band #2 is twice as broad as that needed for band #7. The same holds true even when using 50,000 geometries (δ_*n*,50000_). This situation contrasts with the common procedure of using the same δ for all bands and highlights the importance of resorting to a method capable of choosing a different δ for each band in order to properly reconstruct the full spectrum.

**Table 1:** Optimal Model Parameters for Each of the Bands/Transitions Used to Reconstruct the Spectra in Figure 2

	1	2	3	4	5	6	7	8	9	10
δ_*n*,50000_^a^	0.035	0.039	0.025	0.025	0.026	0.026	0.023	0.024	0.025	0.021
δ_*n*,250_^b^	0.094	0.118	0.074	0.066	0.069	0.072	0.058	0.075	0.071	0.064
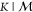 c	6|VVV	2|EVE	3|EEE	2|VVI	3|VVI	2|VVI	3|VVE	3|VVI	3|VVE	3|VVE

a Empirical bandwidths for the target spectrum.

b Empirical bandwidths for the auto-δ spectrum.

c Number of mixtures (*K*) and GMM models  for the GMM-NEA spectrum. VVV: ellipsoidal, varying volume, shape, and orientation; EVE: ellipsoidal, equal volume, and orientation; EEE: ellipsoidal, equal volume, shape, and orientation; VVI: diagonal, varying volume, and shape; VVE: ellipsoidal and equal orientation. For a visualization of these model constraints, check Table 3 and Figure 2 in mclust documentation.^59^

GMM-NEA does not make use of empirical bandwidths, but this method too renders different optimal parameters for each band (Table 1). In this case, the differences are both in the number of mixtures (*K*) and the model constraints . In fact, notice that for band #1 6 components with fully unconstrained covariance matrices (model VVV) are needed, whereas for the rest of bands 2–3 components with more or less constrained covariance matrices suffice. It is worth to digress for a moment to understand the reason why for GMM-NEA the transition dipole moments *M* are used instead of the oscillator strengths *f*. As shown in Figure S1, *f* is a highly right-skewed variable, whereupon a GMM should replicate this skewness with a combination of symmetric (non-skewed) distributions (normals). The model selection procedure (EM algorithm + BIC maximization) would suggest the use of very *skinny* Gaussians to properly model the region of low *f* values, while avoiding the negative *f* region, and then, it would add more and more components with ever *fatter* Gaussians to model the long tail of the *f* distribution. In other words, to model a skewed distribution, GMMs with a higher complexity (components + constraints) are needed. The more complex the model is, the larger the number of parameters to fit becomes. This situation might lead to an overfitting scenario, where there are more parameters to fit than data to use, leading to incorrect density estimations and, in turn, spectral reconstructions. To obtain less complex GMM models and thus avoid overfitting, it is a good practice to transform the skewed variable so that it becomes more symmetrically distributed. There are many transformations that could have been applied (log-transform, Box–Cox, quantiles, etc.), but in this case, we chose a square root transformation, that helps in making the distribution less skewed (Figure S1) and that is physically meaningful .

**Figure 3:**
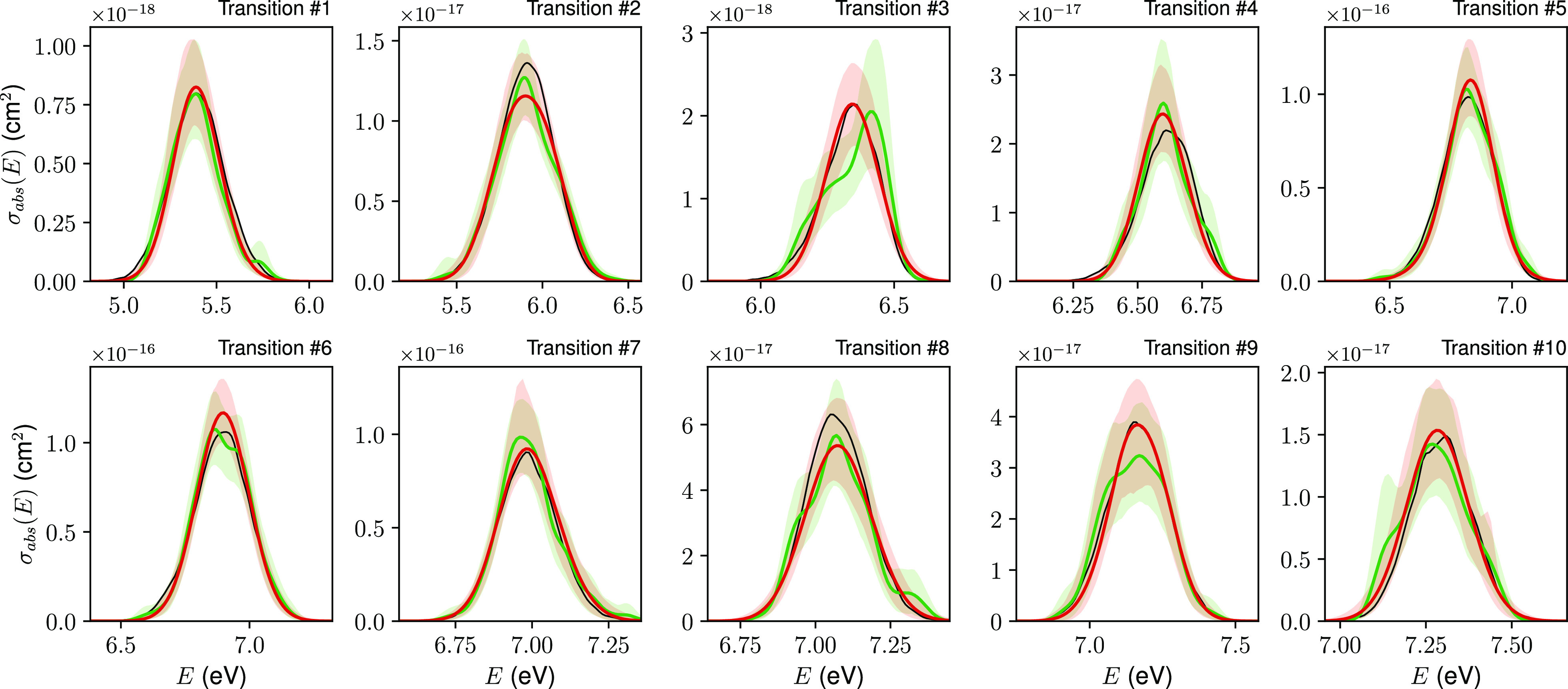
Electronic absorption cross-section spectrum for each of the transitions in benzene reconstructed from 250 geometries using GMM-NEA (red lines) and auto-δ (green lines). The shaded areas represent the reconstruction of 95% CIs. The target spectrum (black lines) is included for comparison purposes.

The spectral reconstruction is not only reasonable for the main absorption features in the full spectrum (Figure 2) but it is as well reliable band to band (Figure 3). In this particular case, it becomes clearer that GMM-NEA seemingly outperforms auto-δ, as its reconstructed bands systematically lay closer to the target ones. This situation is as well observed for derivatives Comp2 and Comp3 (Figures S5 and S6). For these derivatives, though, there are far less available geometries to compute the target spectrum (2000), and thus, its reconstruction using auto-δ still contains too much artificial “vibronic” noise.

### Sample Size and Sampling Bias Effects

**Figure 4:**
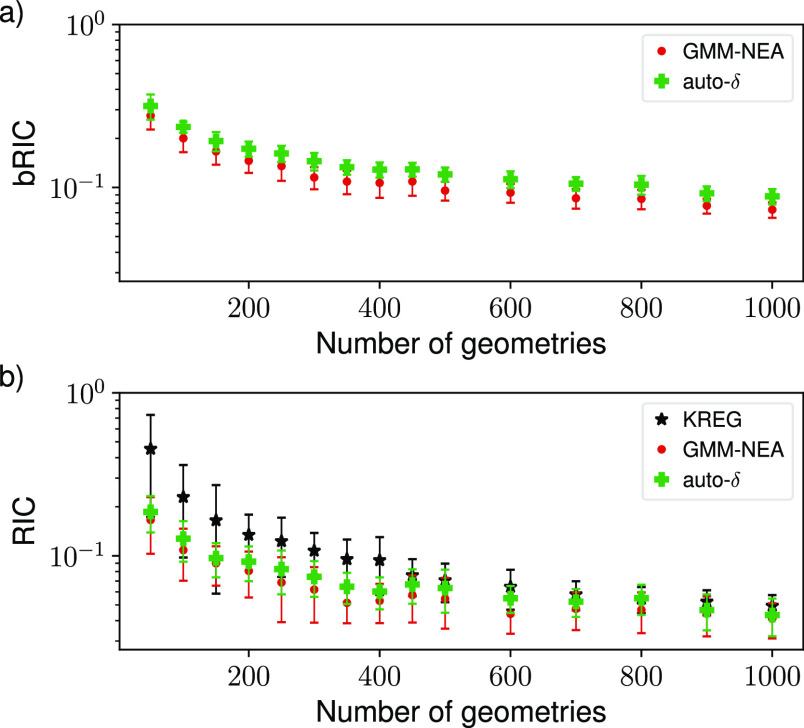
Dependence of (a) bRIC and (b) RIC on the number of geometries used for reconstructing the electronic absorption spectra of benzene using GMM-NEA (red points) and auto-δ (green crosses). The RIC values reported for the spectra reconstructed using the KREG model^30^ (black stars) have been included in (b) for comparison purposes. The markers and error bars indicate the average and standard deviation over 25 independent random draws. The same *y*-scale has been used in both panels for the sake of better comparison.

At this point, one may wonder when the spectral reconstruction is good enough and which of the two proposed methods performs better. This is especially relevant when addressing the computation of absorption spectra requiring high-level quantum chemistry methods (EOM-CCSD, CASPT2, etc.) and/or the study of more complex systems (large number of excited electronic states, spin–orbit coupling, large molecules, solvent effects, etc.). With this in mind, we performed a study to assess the dependence of the electronic spectra on the number of geometries (sample size effect) and on the particular set of geometries (sampling bias effect) used for the reconstruction. The sample size *N*_s_ was varied from 50 up to 1000, and, to account for the sampling bias, 25 different sets of *N*_s_ geometries were randomly sampled from the whole population. For each of these geometry sets, the spectra were reconstructed using GMM-NEA and auto-δ, and, with them, the bRIC was computed using the target bands generated with 50,000 geometries as σ_T,*n*_ (see methods and eq 25). As one would expect, the bRIC decreases when increasing the number of geometries, implying an improvement in the goodness of the spectral reconstruction (Figure 4a). However, the most relevant result of this experiment is that, statistically, GMM-NEA outperforms auto-δ as a reconstruction method since its bRIC values are consistently smaller than those of auto-δ. Actually, we have calculated that for any given set of geometries, GMM-NEA outperforms auto-δ in more than 90% of the cases. This confirms the results observed in Figure 3. Again, analogous results have been obtained for Comp2 and Comp3 (Figures S7 and S8). Nevertheless, notice in Figures S7 and S8 that for a large number of geometries, auto-δ starts to outperform GMM-NEA. However, this can be misleading/artificious, as auto-δ will eventually converge to the target spectrum, which is itself computed using auto-δ with 2000 geometries (a relatively small number). Accordingly, for 600+ geometries, auto-δ has converged to the target (*i.e.*, itself) more than GMM-NEA. Should the number of available geometries for the target spectrum be much larger, this might not be the case.

To compare the spectral reconstruction goodness of GMM-NEA and auto-δ against that of supervised ML algorithms such as the KREG model,^30^ we computed as well in the previous experiment the metric RIC, using the target spectrum generated with 50,000 geometries as σ_*T*_ (see methods and eq 24). Figure 4b displays the results of this calculation and its comparison with the RIC values reported previously for the KREG model applied onto benzene.^30^ Remarkably, in this case, the unsupervised ML models clearly render significantly better reconstructions than those obtained with the KREG model, specially for sample sizes below 400 geometries. The situation is even more drastic for derivative Comp2 (Figure S7). The probable reason for the under performance of the supervised ML model is that the prediction of *f* from molecular descriptors is notoriously difficult.^32,33^

### Active Learning

**Figure 5:**
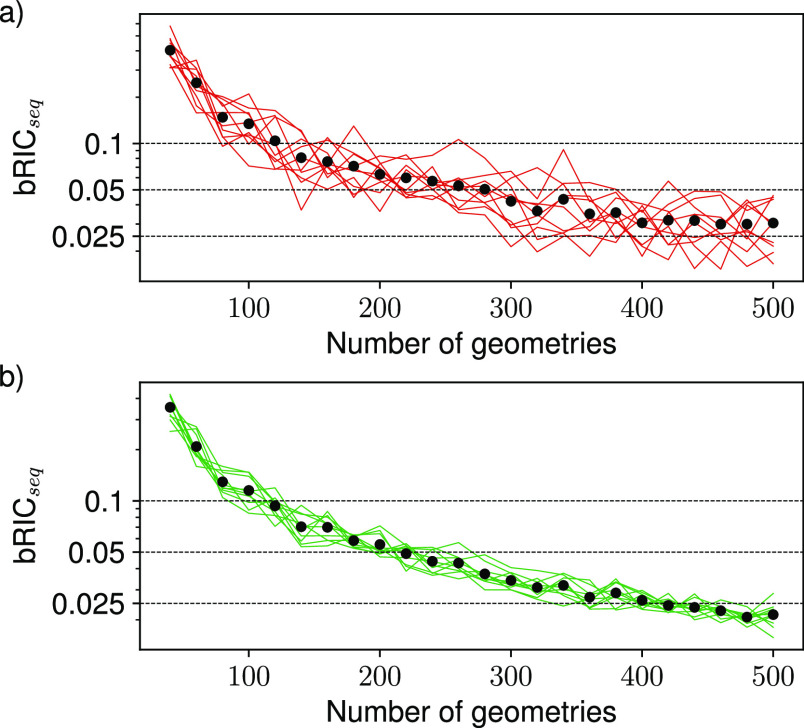
Evolution of bRIC_seq_ with the number of geometries used for reconstructing the electronic absorption spectra of benzene using (a) GMM-NEA and (b) auto-δ. Each line represents an independent experiment. The markers indicate the average over these experiments. The horizontal dotted lines mark the location of bRIC_seq_ = (0.1, 0.05, 0.025).

As we mentioned before, in realistic scenarios, one does not have a target spectrum to compute bRIC, and thus, an “active learning” approach should be followed. With this in mind, we performed another study to assess the applicability of this method. The sample size *N*_s_ was varied from 20 up to 500, adding in each step batches of 20 geometries. For each of these geometry sets, the spectra were reconstructed using GMM-NEA and auto-δ, and, with them, the bRIC_seq_ was computed (see methods and eq 26). To account for the sampling bias, 10 independent experiments were conducted. Notice that there is no value of bRIC_seq_ for 20 geometries, as there are no spectra to compare with (σ_R,*n*_^old^, cf. eq. 26). As we already saw in Figure 4, the addition of more geometries led to more reliable spectra (smaller bRIC), but the improvements became smaller, signaling that the reconstructed spectrum was converging to the target one. Figure 5 reveals this tendency as well, meaning that bRIC_seq_ is a useful metric to ascertain when the reconstructed spectrum following the “active learning” approach has sufficiently converged to the target one, even when we do not have access to it. Analogous results were obtained for Comp2 and Comp3 (Figures S9 and S10).

Of course, it is up to the practitioner to decide when the spectrum has converged sufficiently. If using QM formalisms with a moderate computing time burden (like TD-DFT), one may decide to add more geometries until, for example, bRIC_seq_ < 0.025. For the case of benzene, this threshold condition would entail the selection of around 400 geometries (see Figure 5), whereas for Comp2 and Comp3, it would increase up to 500 geometries (Figures S9 and S10). In situations requiring a much higher computational power (higher-level QM formalisms and/or more complex systems), one could decide to add geometries until bRIC_seq_ < 0.05 or even bRIC_seq_ < 0.1. For benzene, Comp2, and Comp3, the former criterion would entail the selection of around 200–300 geometries (see Figures 5, S9, and S10). Incidentally, the spectra displayed in Figures 2, 3, and S2–S5, which were already quite reliable, were reconstructed using 250 geometries. Accordingly, we believe that the criterion bRIC_seq_ < 0.05 is a good compromise between accuracy and computational burden, but values of bRIC_seq_ < 0.1 or even higher could be reasonable depending on the ultimate goal of the practitioner. Notice that on an individual experiment basis, bRIC_seq_ becomes smaller with the sample size, but it does follow a fluctuating behavior overall for the spectra reconstructed using GMM-NEA. This means that to select the number of geometries in a sequential fashion, it could be recommended to use bRIC_seq_ computed onto the auto-δ spectra.

### Effect of Outliers (The Case of the U6OH Radical)

The presence of outliers (observations significantly differing from the population) in electronic spectrum computations has not been reported till date, as it is not usually looked for nor easily detected. One may argue that any extreme or rare value in either Δ*E* or *M* cannot be considered an outlier but an extreme although totally feasible and fundamental value. Although this is true in most cases, it sometimes happens that the QM computations do not converge to a realistic solution overall when dealing with complex problems and advanced methodologies. For instance, in CASPT2 applications, problems derived from the presence of intruder states, instability of the active space, a reduced number of roots, or differential dynamic correlation are not so infrequent. Although these problems are normally detected and solved by individual analyses of each Δ*E* and *M* calculation or data processing prior to plot generation, efficient algorithms to identify them during the NEA spectral reconstruction stage would surely help the user. In this section, we aim at describing how GMM-NEA or auto-δ can be affected by the presence of outliers.

**Figure 6:**
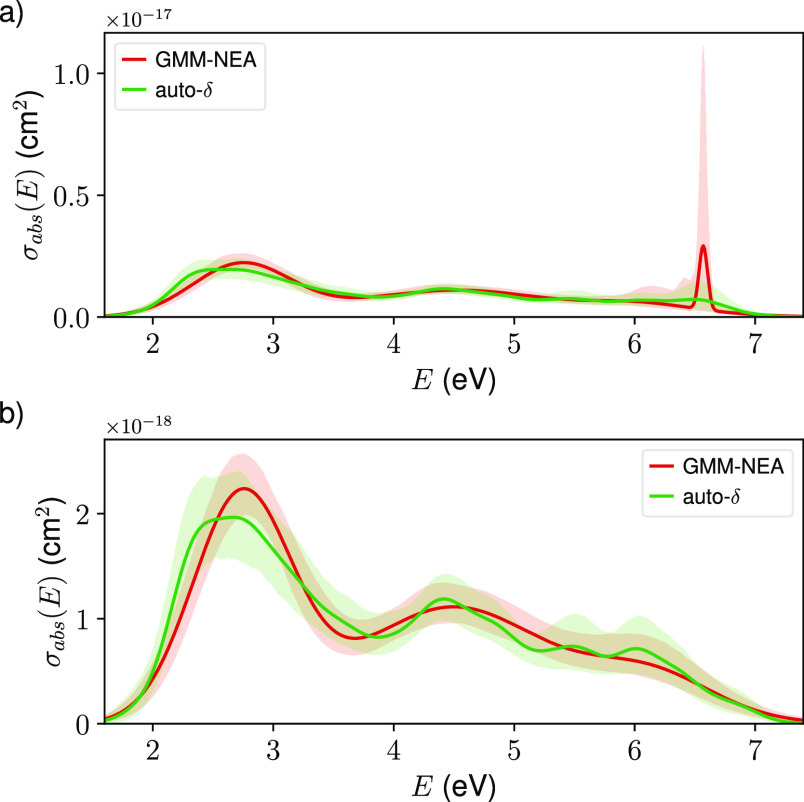
Electronic absorption cross-section spectrum of the U6OH radical reconstructed from 100 geometries using GMM-NEA (red lines) and auto-δ (green lines) in the presence (a) and absence (b) of outliers. The shaded areas represent the reconstruction of 95% CIs.

A particular example that we found during this investigation was that of the U6OH radical. We reconstructed the electronic absorption cross-section spectra using the available cases reported previously^29^ (100 geometries). Both GMM-NEA and auto-δ reconstructed spectra are distorted, specially the former (Figure 6a). The auto-δ spectrum shows a suspiciously large uncertainty around 6.6 eV, whereas the GMM-NEA one portraits what seems a gigantic resonance at the same energy. This suggests that there is an anomaly in one of the transitions in that region. This anomaly can be effectively visualized in the *M* vs Δ*E* plot corresponding to that transition, which shows a clear outlier (Figure S11).

It must be stressed that once a possible outlier/anomaly is detected, the user of the method should revise the corresponding structure and the output of the QM computation for this point, try to interpret the reason why there was this anomaly, and evaluate the relevance of this anomalous point. For example, by tracking the outlier structure in the U6OH radical, we found that it corresponds to the highest root (10th) used for the CASSCF/CASPT2 computations. Although this excited state with large *M* is stabilized in this geometry and it was therefore added in the set of 10 roots during the CASSCF/CASPT2 optimization of the wavefunctions, it is out in the rest of geometries. This indicates that should we properly compute σ_abs_ at this range of energies, the number of roots had to be increased. Nevertheless, for this species of relevance in the field of DNA damage mechanisms by reactive oxygen species, the most interesting range of wavelengths is that of the visible part of the spectrum (<3.3 eV).^29,60^

Once the outlier detection algorithm is applied (see Methodology) and the corresponding geometry removed, the reconstructed spectra show the expected behavior. The uncertainty around 6.6 eV in the auto-δ spectrum is more in agreement with that of the rest of energies, and the GMM-NEA spectrum does not show a false resonance anymore (Figure 6b). An alternative visualization of the effect of the outliers on the GMM-NEA and auto-δ spectra is shown in Figure S12. In this particular case, the effect of the outlier and the outlier itself were easily detected by visual means (Figure S11), but in many other cases, it may not be that trivial. In these situations, the anomalous effect of an undetected outlier could be ascribed to an innocuous spectral feature that, in turn, could lead to wrong conclusions. These results highlight the importance of detecting possible outliers. Nonetheless, the outliers may not have a high leverage in the resulting NEA spectrum. For example, we have as well detected possible outliers in benzene, but, in this case, the changes in the spectra were barely noticeable, and therefore, they are irrelevant.

Finally, whereas the FDR method works adequately, it might not be necessarily the best method to detect outliers in the context of QM calculations, specially when dealing with high-dimensional data (many tens of transitions), and other anomaly detection algorithms could perform better. Although relevant, attempting a serious and comprehensive comparison of anomaly detection methods in this context is beyond the scope of this publication, which is mainly focused on the use of GMMs for spectral reconstruction.

### Photolysis Rates in HgBrOOH

Once the performance of the proposed methods has been assessed under diverse conditions, we will apply it to a problem of interest. Namely, the accurate determination of the photolysis rate of an oxidized Hg species, HgBrOOH, present in the Earth’s atmosphere and involved in the planetary distribution of this metal.^5,58^ The photolysis rate *J* is defined as
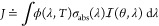
27where ϕ(λ,*T*) is the photolysis quantum yield as a function of the wavelength and temperature, σ_abs_(λ) is the absorption cross-section spectrum, and  is the solar spectral actinic flux (in quanta s^–1^ cm^–2^ nm^–1^) at the altitude of interest as a function of solar zenith angle θ and the wavelength. Thus, the correct reconstruction of the absorption spectrum σ_abs_(λ) is fundamental for a precise estimation of the photolysis rate *J*.

**Figure 7:**
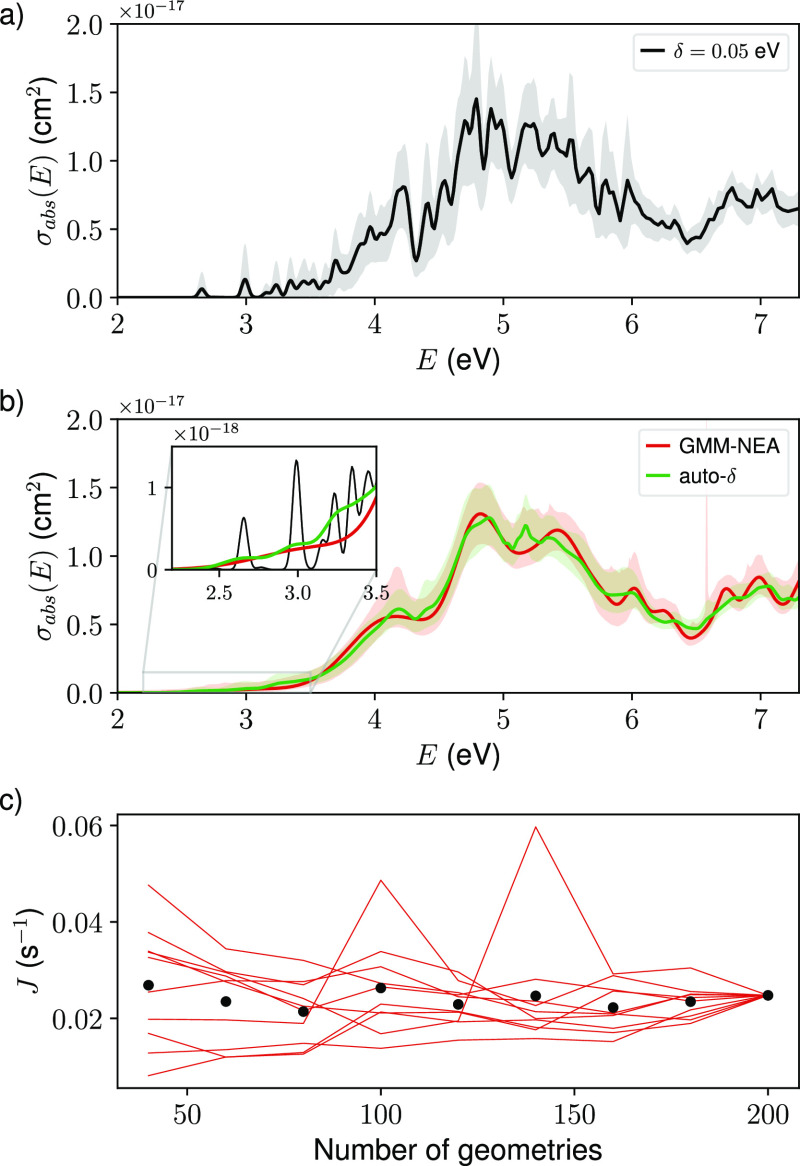
(a) Electronic absorption cross-section spectrum of HgBrOOH reconstructed from 200 geometries using a unique empirical bandwidth for all transition (δ = 0.05 eV). (b) Same as (a) but using GMM-NEA (red lines) and auto-δ (green lines). The shaded areas in (a,b) represent the reconstruction of 95% CIs. The inset in (b) details the contribution of three spectra in the region of maximum solar radiation. (c) Evolution of the photolysis rate *J* with the number of geometries used for reconstructing the electronic absorption spectra of HgBrOOH using GMM-NEA. Each line represents an independent experiment. The markers indicate the average over these experiments.

The reconstructed spectrum for this compound was already reported,^58^ where an empirical bandwidth δ = 0.05 eV was applied to all bands (Figure 7a). This choice of δ resulted in the presence of apparently strong and quite resolved bands around 2.6 and 3 eV. The absorption at these bands could play a role in the photolysis reaction of this compound, as they overlap with a region of strong solar radiation (Figure S13). Nevertheless, the reconstructed spectra using both auto-δ and GMM-NEA unveil that there is indeed absorbance in that region but that the bands are not as resolved as previously reported (Figure 7b).

One may wonder whether this change in the spectral shape is followed by an important change in the photolysis rate *J*. To assess this extent, we compared the *J* obtained with the three spectra (δ = 0.05 eV, auto-δ, and GMM-NEA). For simplicity, we assumed in eq 27 that ϕ(λ,*T*) = 1 and used spectral actinic flux  calculated by using the “quick TUV calculator” tool^61^ assuming an altitude of 13 km from the sea level (mean of the troposphere) and normal solar incidence (θ = 0). The resulting solar spectrum is displayed in Figure S13. The photolysis rates *J* obtained under these conditions were 0.025, 0.026, and 0.025 s^–1^ for δ = 0.05 eV, auto-δ, and GMM-NEA, respectively. Remarkably, the method to reconstruct the absorption spectrum has not much influence on the computed photolysis rate as long the value of δ is sensibly chosen. In this sense, many times a single geometry (the ground-state equilibrium structure) is used to reconstruct electronic spectra. Using a unique geometry may lead to wild errors in the determination of the photolysis rates. For example, the exclusive use of the optimized geometry for this compound leads to large variations in the computed *J* as a function of the empirical bandwidth δ (Figure S14). When using a single geometry, it is impossible to know beforehand which is the optimal bandwidth δ, and thus, there will always be a large uncertainty in the determination of the photolysis rates. This result highlights the need of using a representative sample of geometries to reconstruct electronic spectra.

Following with this line of reasoning, another relevant aspect is to understand how the number of sampled geometries affects the determination of the photolysis rate. With this in mind, we ran an experiment analogous to the one previously described to assess the usefulness of the “active learning” approach. Namely, the sample size *N*_s_ was varied from 20 up to 200, adding in each step batches of 20 geometries. For each of these geometries sets, the spectra were reconstructed using GMM-NEA and auto-δ, and, with them, *J* was computed. To account for the sampling bias, 10 independent experiments were conducted. The first thing to notice is that the value of *J* is importantly affected by sampling bias, specially when using a small number of geometries (Figure 7c). It might seem that the sampling bias is reduced for the largest sample sizes, and it is indeed, but this reduction is somewhat fictitious, as there are only 200 geometries to sample from. As a consequence, the 10 independently drawn samples will be very similar when sampling more than 150 of them, resulting in very similar *J* values. In any case, it is true that *J* converges, on an individual experiment basis, toward a constant value as the sample size increases, but it does follow a fluctuating behavior.

Overall, the range of *J* values obtained herein for HgBrOOH reinforces the conclusions obtained in our previous investigations on the significant role of solar radiation to photoreduce this compound (and other oxidized Hg species) to elementary Hg.^5,58^ As a final note, we comment that the bRIC_seq_ in this compound is barely at a level of 0.1 for the auto-δ spectrum generated with 200 geometries (Figure S15). This indicates that if higher accuracy is demanded in future studies, for instance, to discern among competitive processes, we should work in the direction of decreasing this value by increasing the sample size.

## Conclusions

In this work, we have developed and tested a new approach to reconstruct NEA spectra based on the use of GMMs that circumvent the use of phenomenological broadenings and, in turn, the selection of a bandwidth δ altogether. The key for this approach is to mathematically transform the conventional equation for the reconstruction of NEA spectra (eq 1) to express it in terms of the GMM parameters that model the distribution of the pairs {Δ*E*_*i*_,*M*_*i*_}_*i*=1,...,*Ns*_ for each transition (eq 22). Globally, GMM-NEA systematically outperforms both the KREG and KDE models (auto-δ herein) in reconstructing both the full spectrum and the different transition band shapes overall for small datasets (less than 400 geometries). Although choosing an adequate δ, either manually or using auto-δ, is an easier and less time-consuming task (see the Supporting Information), the benefits of GMM-NEA are sufficiently relevant as to choose the former over the latter overall when the computational bottleneck is clearly in the QM calculations. In addition, we have proved the importance of detecting anomalous QM computations leading to inaccurate values of the oscillator strength for certain geometries and transitions. These outliers, if undetected, may lead to heavy distortions both in the NEA spectra and their CIs, specially for those reconstructed using GMM-NEA. In contrast, when performing computations with the reconstructed spectra, like inferring the photolysis rate, GMM-NEA leads to virtually the same results as auto-δ or a “manual” selection of bandwidths (as long as δ is chosen sensibly), probably because it involves an integration over wavelengths that washes out the fine details of the NEA spectra.

Another great advantage of GMM-NEA (and other unsupervised ML methods) with respect to supervised ML algorithms such as NNs or the KREG model for the reconstruction of NEA spectra is that it does not rely anymore on the critical step of defining adequate molecular descriptors or on the difficulty of mapping the molecular structure space onto the chemical properties’ space. This is particularly relevant for the incorporation of a solvent, embedding, and/or environment effects (proteins, nucleic acids, surfaces, interfaces, etc.) beyond the continuum solvation model.^29^ In these complex systems, the number of molecular descriptors increases dramatically and, more importantly, the values of Δ*E* and *f* do not depend exclusively on the molecular geometry. Finally, the methodology presented in this article should be fully compatible with the strategy to finding the optimal choice of the nuclear ensemble geometries recently reported by Sršeň and Slavíček.^27^ In this sense, the combination of GMM-NEA and this method could lead to a fairly accurate reconstruction of NEA spectra resorting to the QM computation of Δ*E* and *f* for just tens of geometries.

## Supporting Information Available

Study on the computation complexity of auto-δ and GMM-NEA; optimal model parameters for each of the bands/transitions used to reconstruct the spectra; histograms of Δ*E*, *f*, and *M* for a selection of transitions in benzene; execution times of auto-δ and GMM-NEA as a function of the number of geometries and number of states; NEA full-spectrum and band cross sections for Comp2 and Comp3 using GMM-NEA and auto-δ; variation of bRIC and RIC with the number of geometries for Comp2 and Comp3 using GMM-NEA, auto-δ, and the KREG model; KREG model predictions vs. ground truth for Δ*E*_*n*_ and *f*_*n*_ in benzene; variation of bRIC_seq_ with the number of geometries for Comp2 and Comp3 using GMM-NEA and auto-δ; 2D plot of *M*_9_ versus Δ*E*_9_ in the U6OH radical; overlap between the solar spectrum and the HgBrOOH absorption cross-section spectrum; variation of the HgBrOOH photolysis rate *J* with δ; and variation of bRIC_seq_ with the number of geometries for HgBrOOH using GMM-NEA and auto-δ (PDF)

## Accession Codes

A fully functional and flexible version of the code to reconstruct the NEA spectra using both auto-δ and GMM-NEA is available in the GitHub repositories https://github.com/lucerlab/GMM-NEA and/or https://github.com/qcexval/GMM-NEA under an LGPL-2.1 License. The current implementation can run in both Windows and Linux environments (it has not been tried in MacOS, but it should work as well).
