# Supplementary material for: Reconstruction of Nuclear Ensemble Approach Electronic Spectra Using Probabilistic Machine Learning
Source: J Chem Theory Comput. 2022 Apr 28;18(5):3052–64. doi: 10.1021/acs.jctc.2c00004 (PMC9097286; doi:10.1021/acs.jctc.2c00004)
Supplement: Supplementary file 1 — ct2c00004_si_001.pdf [file ct2c00004_si_001.pdf]

SUPPLEMENTARY MATERIALS

for

Reconstruction of Nuclear Ensemble Approach Electronic Spectra using Probabilistic Machine Learning

Luis Cerdán* and Daniel Roca-Sanjuán*

Institut de Ciència Molecular, Universitat de València, València 46071, Spain

E-mail: lcerdanphd@gmail.com; daniel.roca@uv.es

Supplementary text: Execution time

We have carried out an execution time study to compare the computation burden of auto-δ and GMM-NEA (see Fig. S2). To run this experiment a conventional Windows 10 (64-bits) laptop with an AMD Rodeon processor (7th Gen A12-9720P) with 4 available threads, a clock speed of 2.7 GHz, and 8Gb of RAM was used. In our current implementation, each state/transition is processed by a single thread in a multi-threading fashion. This means that for this machine, 4 states/transitions could be computed simultaneously in parallel. Accordingly, running this implementation in a cluster with many more available threads would reduce the computation time significantly. In particular, the computation time would scale as $\mathcal{O}(1/t)$ with the number of threads t, although using more threads than states would be useless.

As a general comment, computing auto-δ spectra is significantly faster than the computation of GMM-NEA ones, especially when the CI is not computed. In this sense, GMM-NEA can be between 1 and 3 orders of magnitude slower than auto-δ, overall for a large number of geometries (Fig. S2). But, interestingly, when calculating the CI, auto-δ is only 1 order of magnitude faster than GMM-NEA. The complexity and burden of the computation of auto-δ spectra increases as $\mathcal{O}(n)$ where n can be either the number of geometries or the number of states. The calculation with the CI is between 2 and 3 orders of magnitude slower irrespective of the number of geometries or states, which is coincidental with the fact that the implementation repeats the computation 999 times (once per bootstrap replica). This indicates that the actual computation of the spectrum (eq. (1)) is the most demanding step in auto-δ.

The complexity of the calculation of GMM-NEA spectra is more complicated to determine and explain (Fig. S2). The number of parameters to fit in a GMM is $p = K \cdot D_f - 1$, where K is the number of mixtures, and $D_f = 6$ are the degrees of freedom or the number of parameters to fit for a single bivariate normal (2 means, the 3 elements of the covariance matrix, and the mixing coefficient). Assuming at least 5 observations per parameter to avoid overfitting, the maximum number of parameters to consider would be $p = n/5$. Thus, the maximum number of mixtures would be $K_{max} = \lfloor (n/5 + 1)/D_f \rfloor$. For a small number of geometries ($n < 100$), K is restricted to 2 or 3 maximum. For $n = 300$ geometries, $K_{max} = 10$, and for $n = 1000$ up to 33 mixtures can be used. To alleviate the computation burden, our implementation limits the maximum number of mixtures to $K_{max} = 10$, which is more than enough (the most complex GMM that we have observed in all datasets has 8 mixtures). Thus, for $n < 100$ and $n > 300$ geoms, the computation complexity scales as $\mathcal{O}(n)$ due to an increase in the number of geometries only. Whereas for $100 < n < 300$, the complexity scales as $\mathcal{O}(n^3)$ not only due to an increase in the number of geometries but due to an increase in the number of mixtures to use. Finally, it increases as $\mathcal{O}(n)$ with the number of states, as the number of mixtures is always the same. Similar to auto-δ, the calculation of the CI is 1 or 2 orders of magnitude slower, but the difference in execution time between computing the CI and not is reduced with the number of geometries. This indicates that the most demanding step in GMM-NEA is the model selection process, not the computation of the spectra (eq. (22)).

Suppementary table

Table S1: Optimal model parameters for each of the bands/transitions used to reconstruct the spectra in Figs. S3 and S4.

	Comp2			Comp3		
Transition #	$\delta_{n,2000}{}^a$	$\delta_{n,250}{}^b$	$K\|\mathcal{M}^c$	$\delta_{n,2000}{}^a$	$\delta_{n,250}{}^b$	$K\|\mathcal{M}^c$
1	0.096	0.062	2|EEE	0.141	0.094	2|EII
2	0.113	0.072	2|VEI	0.129	0.088	2|VVV
3	0.095	0.065	2|EEE	0.118	0.082	2|EEI
4	0.068	0.05	2|VII	0.111	0.073	2|EII
5	0.072	0.047	4|EEI	0.098	0.069	2|EEE
6	0.061	0.046	2|EVI	0.087	0.057	2|VII
7	0.08	0.053	2|VVI	0.078	0.053	2|VVI
8	0.074	0.052	2|EEV	0.068	0.044	2|EEI
9	0.051	0.049	2|VVI	0.055	0.043	2|EVI
10	0.078	0.051	2|VVI	0.069	0.046	2|EII
11	0.093	0.059	3|VVI	0.075	0.056	2|VEV
12	0.076	0.059	2|EVI	0.079	0.058	2|EEI
13	0.086	0.056	3|EVI	0.074	0.056	2|EEI
14	0.085	0.056	3|VEE	0.092	0.06	2|VII
15	0.073	0.057	2|EVI	0.091	0.063	3|EVI
16	0.086	0.056	2|EEE	0.082	0.063	2|VVI
17	0.079	0.057	2|EVI	0.08	0.06	2|VVI
18	0.103	0.061	2|VEI	0.068	0.06	2|VVI
19	0.105	0.063	3|VEV	0.079	0.057	2|VVI
20	0.091	0.061	2|VVI	0.072	0.055	2|EEI
21	0.079	0.054	2|EVI	0.075	0.053	2|EVI
22	0.07	0.048	2|EVI	0.069	0.052	2|VEI
23	0.074	0.05	3|EVI	0.069	0.049	3|VVE
24	0.075	0.049	2|VVI	0.058	0.047	2|EEI
25	0.065	0.047	2|VVI	0.066	0.044	2|EII
26	0.067	0.047	2|VVI	0.064	0.046	2|EEI
27	0.067	0.046	2|EEI	0.062	0.044	2|EEI
28	0.069	0.047	2|EVI	0.065	0.043	2|EVE
29	0.075	0.045	2|EVI	0.058	0.041	2|EVI
30	0.064	0.046	2|EEI	0.056	0.039	2|EVI

[a] Empirical bandwidths for target spectrum; [b] Empirical bandwidths for auto-δ spectrum. [c] Number of mixtures (K) and GMM models ($\mathcal{M}$) for GMM-NEA spectrum. EII: spherical, equal volume; VII: spherical, unequal volume; EEI: diagonal, equal volume and shape; VEI: diagonal, varying volume, equal shape; EVI: diagonal, equal volume, varying shape; VVI: diagonal, varying volume and shape; EEE: ellipsoidal, equal volume, shape, and orientation; VEE: ellipsoidal, equal shape and orientation; EVE: ellipsoidal, equal volume and orientation; VVE: ellipsoidal, equal orientation; EEV: ellipsoidal, equal volume and equal shape; VEV: ellipsoidal, equal shape; EVV: ellipsoidal, equal volume; VVV: ellipsoidal, varying volume, shape, and orientation.

Supplementary figures

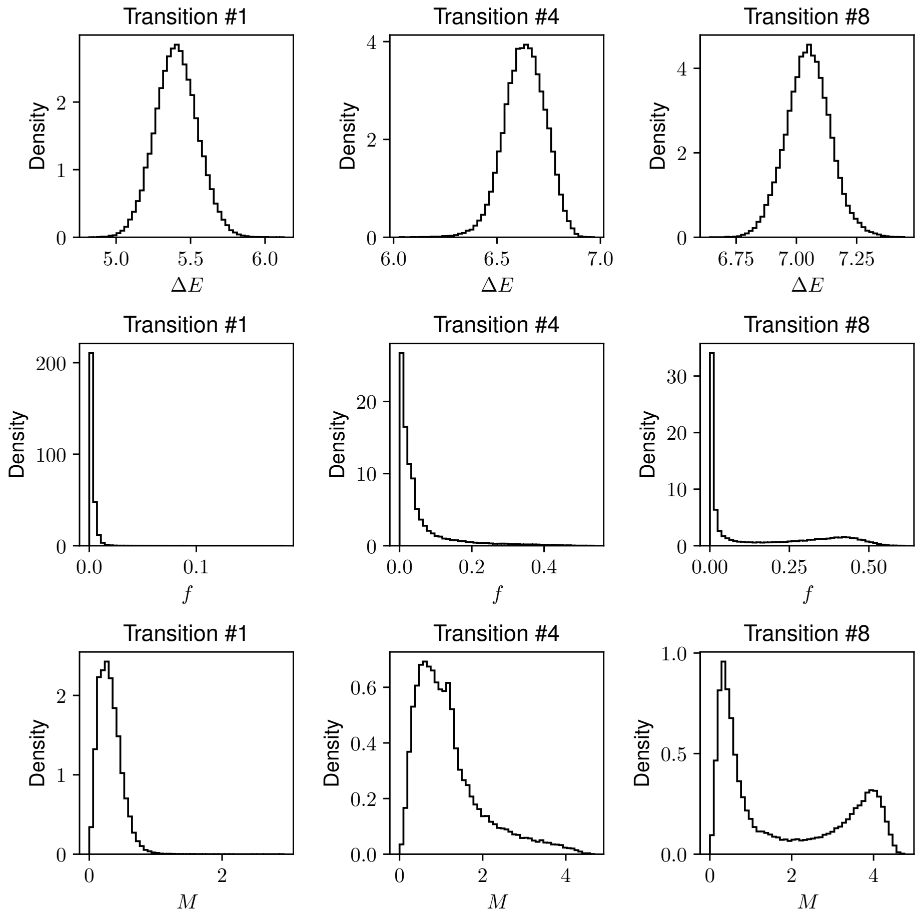

Figure S1: Distribution of values of the vertical transition energies ΔE, oscillator strengths f, and transition dipole moments M for a selection of electronic transitions for benzene.

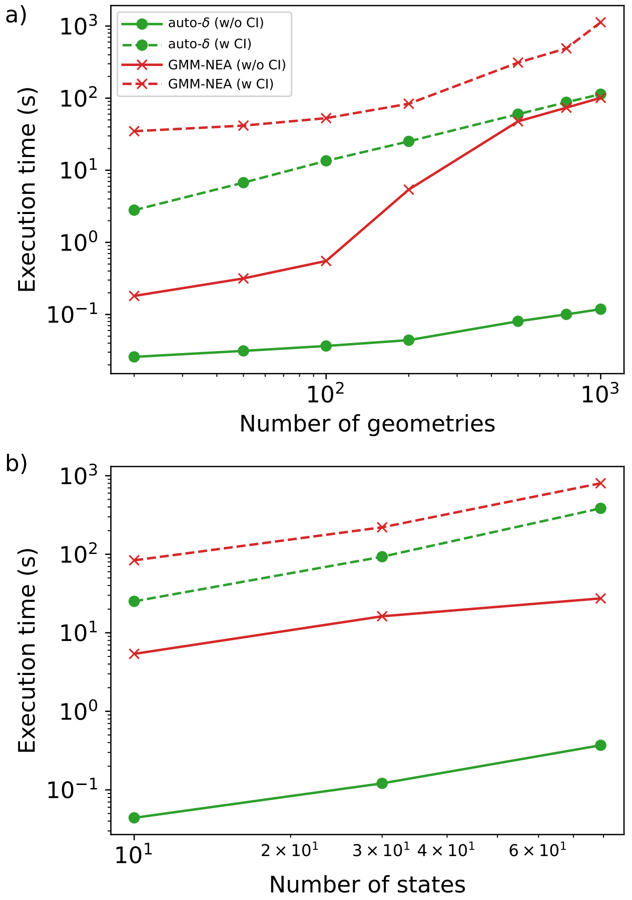

Figure S2: Execution time of auto-δ (crosses) and GMM-NEA (points) as a function of a) number of geometries (for benzene) and b) number of states (for 250 geometries). The dashed and solid lines display, respectively the execution times computing the confidence intervals (CI) or not. The markers indicate the average over 5 independent experiments. The error bars are not included, as they are smaller than the markers.

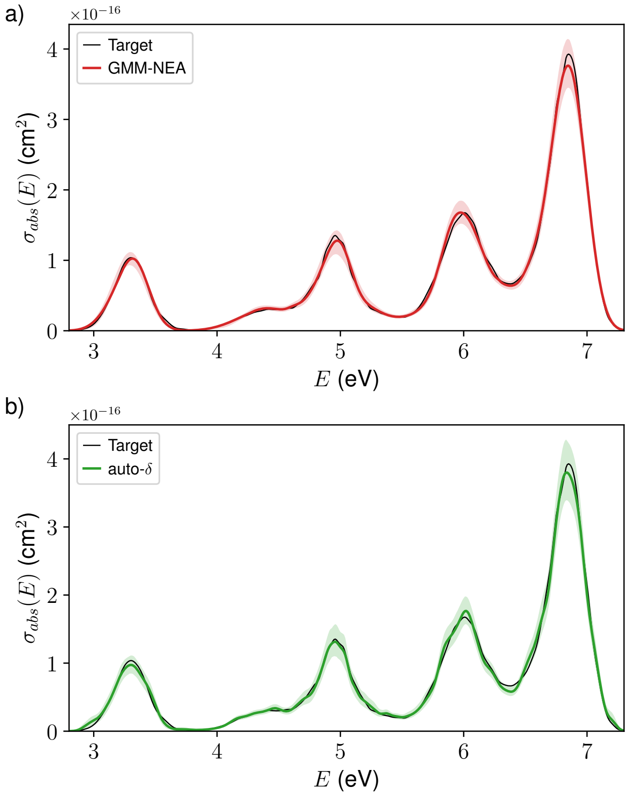

Figure S3: Electronic absorption cross section spectrum of Comp2 reconstructed from 250 geometries using a) GMM-NEA and b) auto-δ. The shaded areas represent the reconstruction 95% CI. The target spectrum (black lines) is included for comparison purposes.

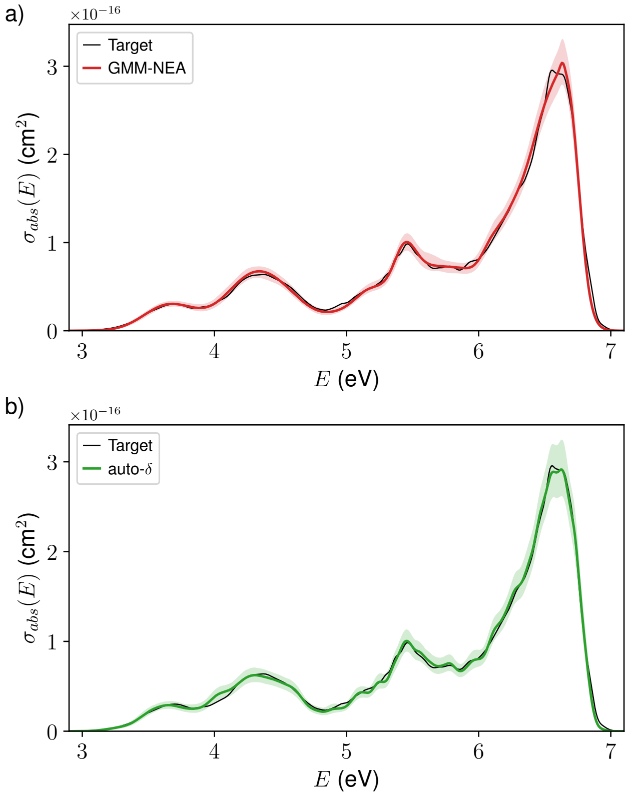

Figure S4: Electronic absorption cross section spectrum of Comp3 reconstructed from 250 geometries using a) GMM-NEA and b) auto-δ. The shaded areas represent the reconstruction 95% CI. The target spectrum (black lines) is included for comparison purposes.

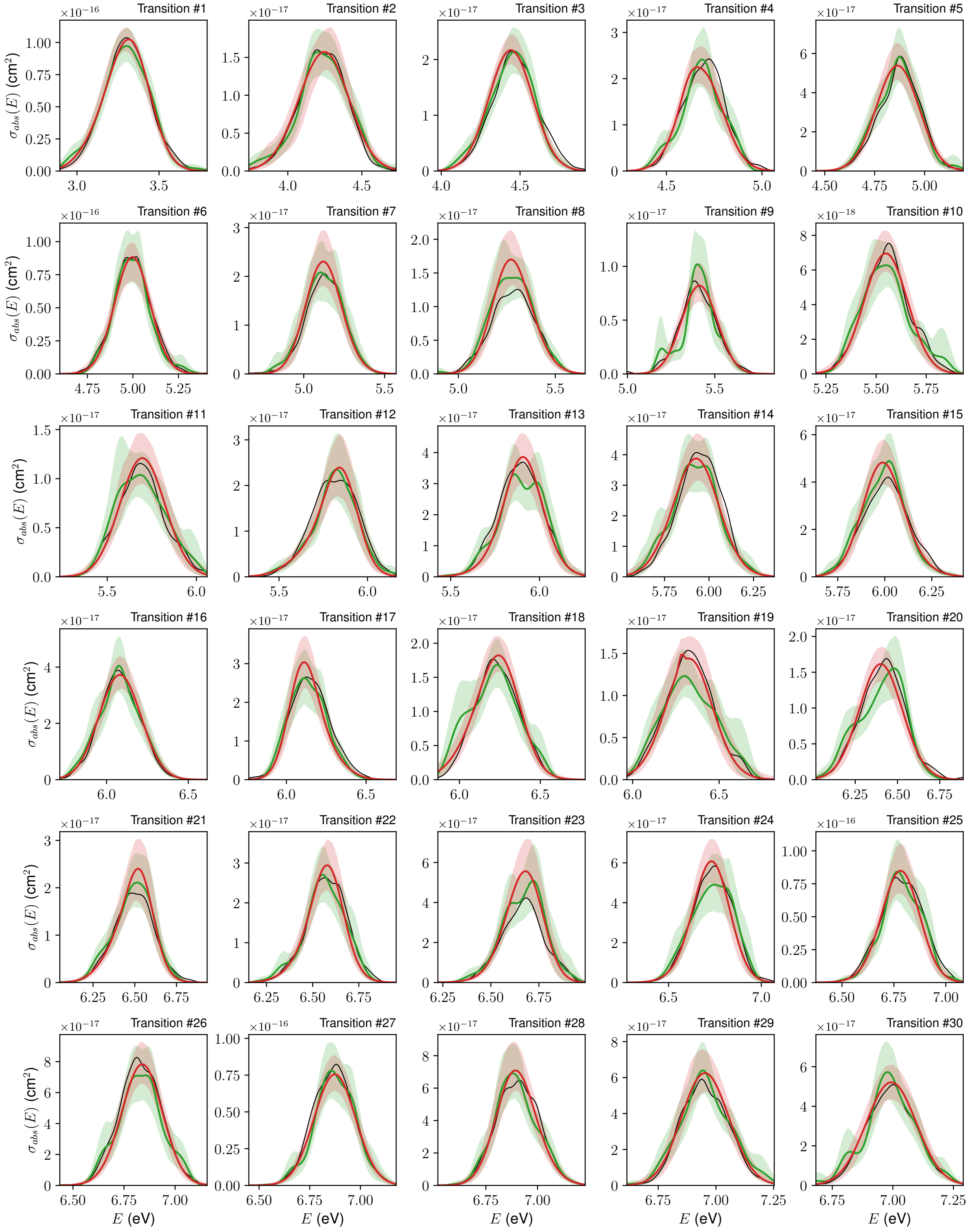

Figure S5: Electronic absorption cross section spectrum for each of the transitions in Comp2 reconstructed from 250 geometries using GMM-NEA (red lines) and auto-δ (green lines). The shaded areas represent the reconstruction 95% CI. The target spectrum (black lines) is included for comparison purposes.

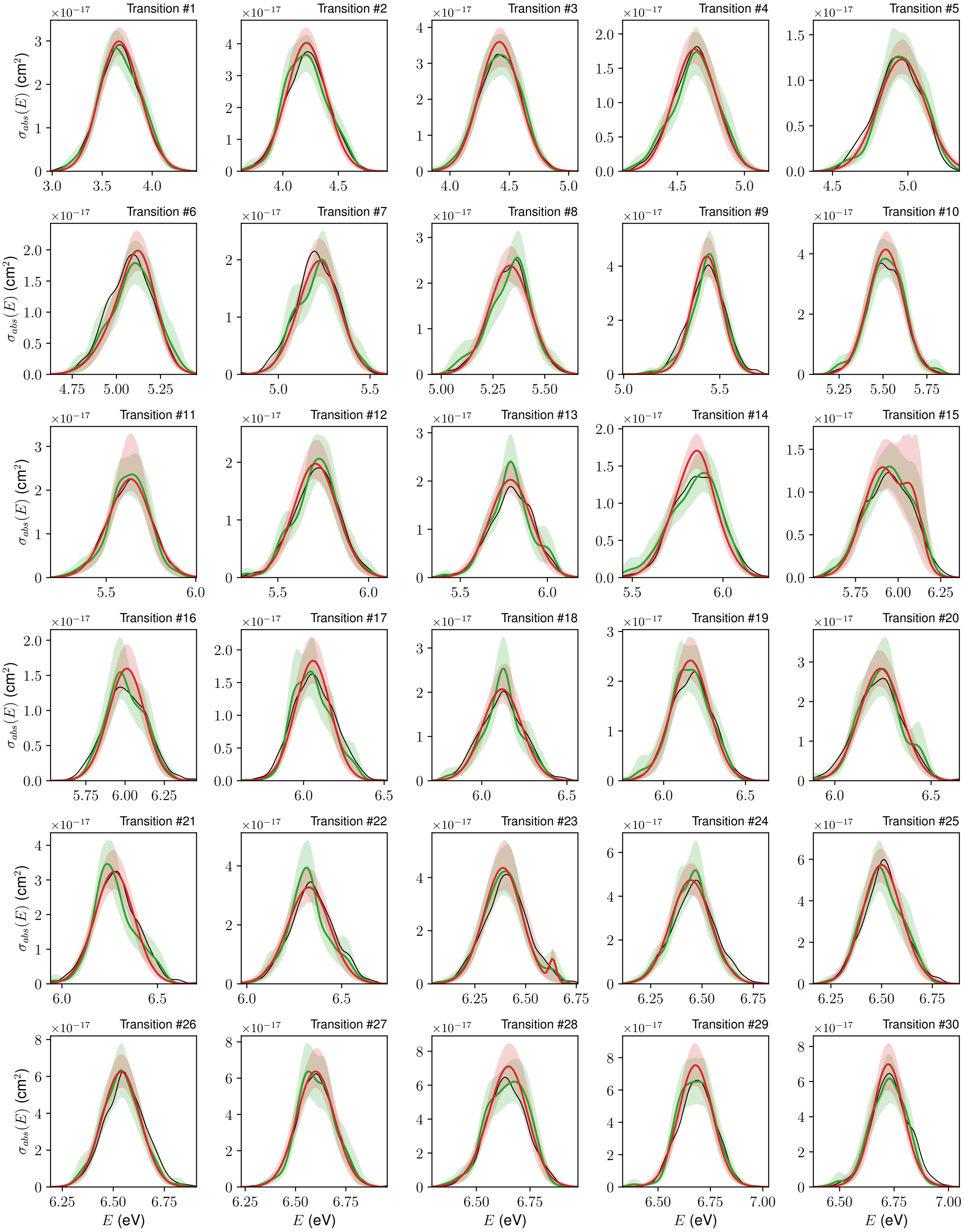

Figure S6: Electronic absorption cross section spectrum for each of the transitions in Comp3 reconstructed from 250 geometries using GMM-NEA (red lines) and auto-δ (green lines). The shaded areas represent the reconstruction 95% CI. The target spectrum (black lines) is included for comparison purposes.

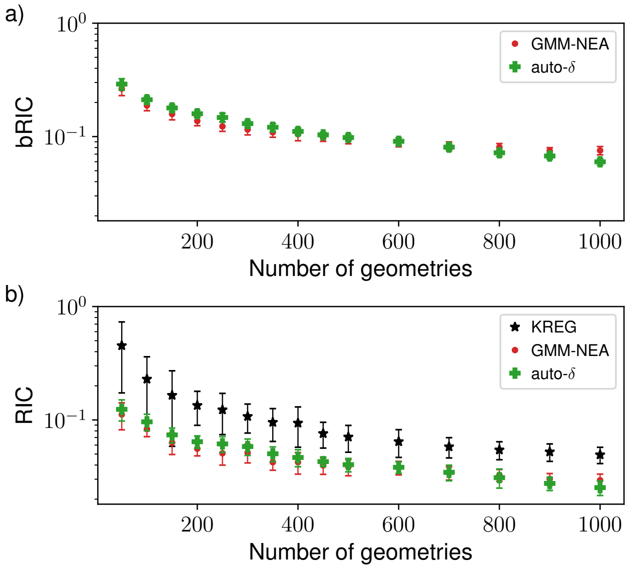

Figure S7: Dependence of a) $bRIC$ and b) RIC on the number of geometries used for reconstructing the electronic absorption spectra of Comp2 using GMM-NEA (red points) and auto-δ (green crosses). The RIC values reported for the spectra reconstructed using the KREG model (black stars) have been included in b) for comparison purposes. The markers and error bars indicate the average and standard deviation over 25 independent random draws. The same y-scale has been used in both panels for the sake of better comparison.

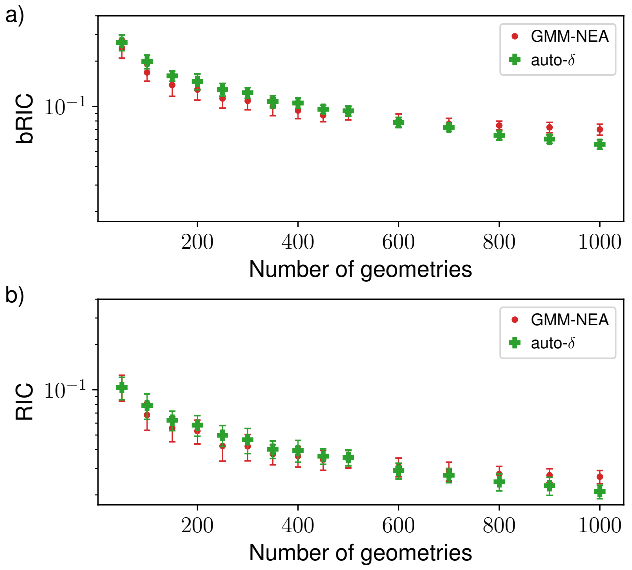

Figure S8: Dependence of a) $bRIC$ and b) RIC on the number of geometries used for reconstructing the electronic absorption spectra of Comp3 using GMM-NEA (red points) and auto-δ (green crosses). The markers and error bars indicate the average and standard deviation over 25 independent random draws. The same y-scale has been used in both panels for the sake of better comparison.

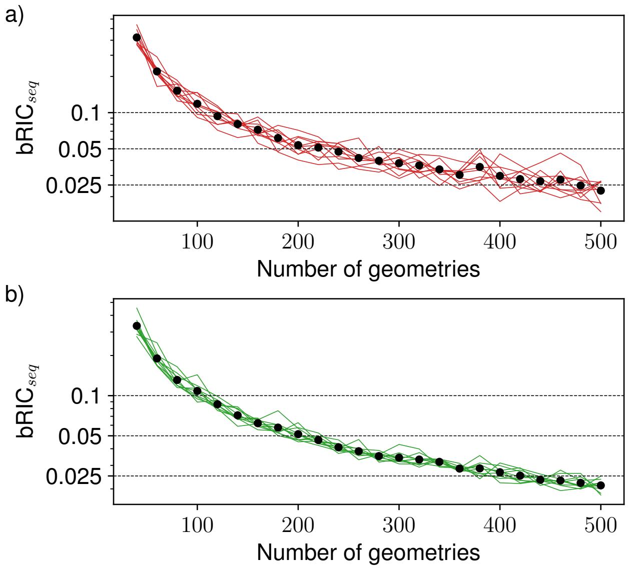

Figure S9: Evolution of $bRIC_{seq}$ with the number of geometries used for reconstructing the electronic absorption spectra of Comp2 using a) GMM-NEA and b) auto-δ. Each line represents an independent experiment. The markers indicate the average over those experiments. The horizontal dotted lines mark the location of $bRIC_{seq} = (0.1, 0.05, 0.025)$.

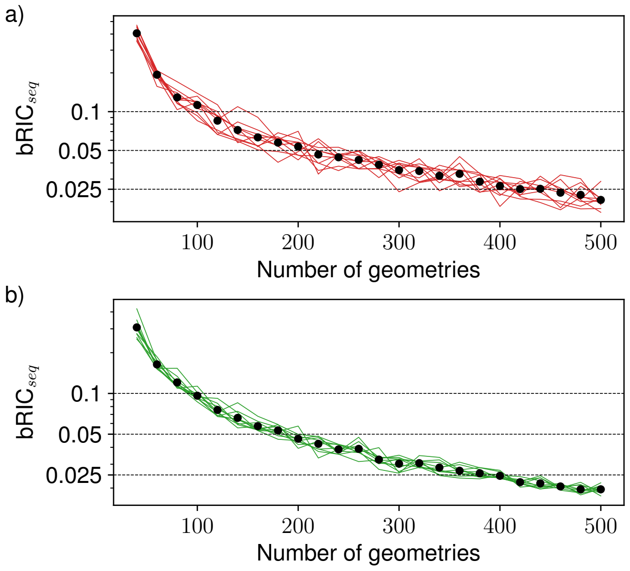

Figure S10: Evolution of $bRIC_{seq}$ with the number of geometries used for reconstructing the electronic absorption spectra of Comp3 using a) GMM-NEA and b) auto-δ. Each line represents an independent experiment. The markers indicate the average over those experiments. The horizontal dotted lines mark the location of $bRIC_{seq} = (0.1, 0.05, 0.025)$.

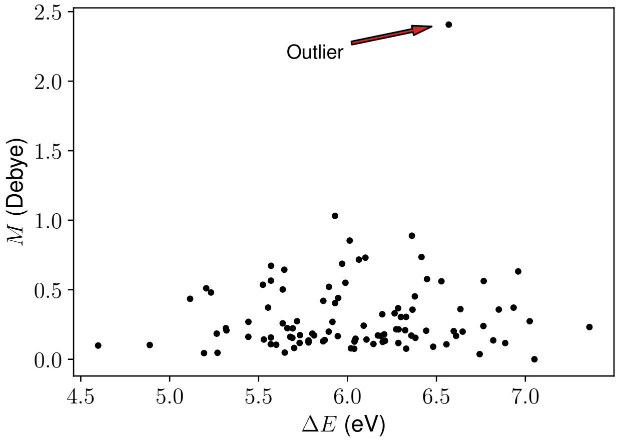

Figure S11: 2D plot of transition dipole moment M versus vertical excitation energy ΔE for transition #9 in U6OH radical.

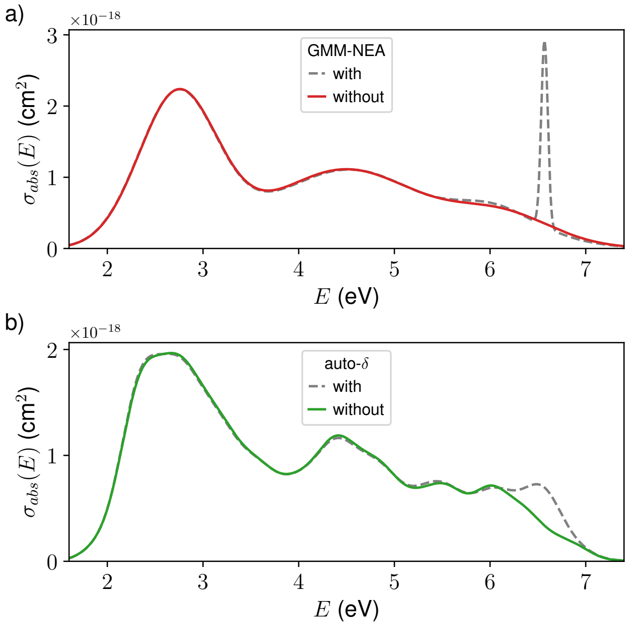

Figure S12: Electronic absorption cross section spectrum of U6OH radical reconstructed from 100 geometries using a) GMM-NEA and b) auto-δ in the presence (dashed lines) and absence (solid lines) of outliers.

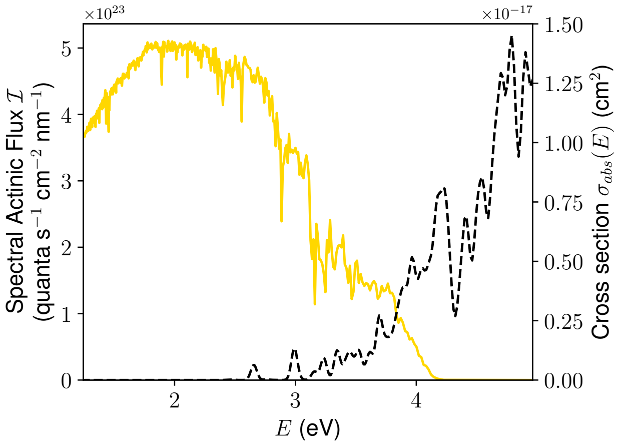

Figure S13: Overlap between the Solar spectrum (yellow solid line) under normal incidence at the troposphere (13 km from sea-level) and the electronic absorption cross section spectrum (dashed line) of HgBrOOH reconstructed from 200 geometries using a unique empirical bandwidth for all transitions ($\delta = 0.05$ eV). Left axis: scale for solar flux; Right axis: scale for cross section).

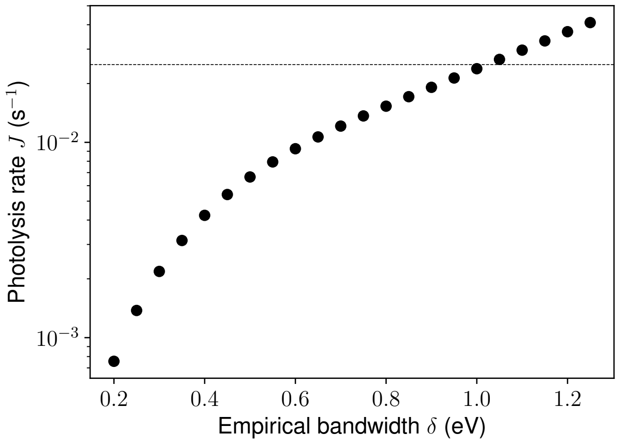

Figure S14: Evolution of Photolysis rate J with the empirical bandwidth δ used for reconstructing the electronic absorption spectra of HgBrOOH using the optimized geometry. The horizontal line marks the position of J as calculated for the spectrum reconstructed using 200 geometries and GMM-NEA.

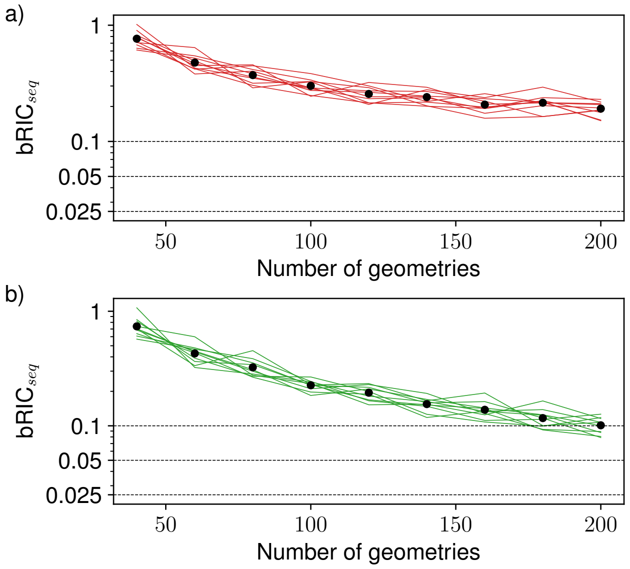

Figure S15: Evolution of $bRIC_{seq}$ with the number of geometries used for reconstructing the electronic absorption spectra of HgBrOOH using a) GMM-NEA and b) auto-δ. Each line represents an independent experiment. The markers indicate the average over those experiments. The horizontal dotted lines mark the location of $bRIC_{seq} = (0.1, 0.05, 0.025)$.
